# The combination of DNA methylome and transcriptome revealed the intergenerational inheritance on the influence of advanced maternal age

**DOI:** 10.1002/ctm2.990

**Published:** 2022-09-14

**Authors:** Lingyue Hua, Wei Chen, Yan Meng, Meng Qin, Zhiqiang Yan, Rui Yang, Qiang Liu, Yuan Wei, Yangyu Zhao, Liying Yan, Jie Qiao

**Affiliations:** ^1^ Center for Reproductive Medicine Department of Obstetrics and Gynecology Peking University Third Hospital Beijing China; ^2^ National Clinical Research Center for Obstetrics and Gynecology, Peking University Third Hospital Beijing China; ^3^ Key Laboratory of Assisted Reproduction, Peking University Ministry of Education Beijing China; ^4^ Beijing Key Laboratory of Reproductive Endocrinology and Assisted Reproductive Technology Beijing China; ^5^ Department of Obstetrics and Gynecology Beijing Jishuitan Hospital, Fourth Clinical College of Peking University Beijing China; ^6^ Department of Obstetrics and Gynecology Peking University Third Hospital Beijing China; ^7^ National Center for Healthcare Quality Management in Obstetrics Beijing China; ^8^ Beijing Advanced Innovation Center for Genomics Beijing China; ^9^ Peking‐Tsinghua Center for Life Sciences Peking University Beijing China; ^10^ Research Units of Comprehensive Diagnosis and Treatment of Oocyte Maturation Arrest, Chinese Academy of Medical Sciences Beijing China

**Keywords:** advanced maternal age, DNA methylation, intergenerational inheritance, transcriptome

## Abstract

**Background:**

The number of women delivering at advanced maternal age (AMA; > = 35) continuously increases in developed and high‐income countries. Large cohort studies have associated AMA with increased risks of various pregnancy complications and adverse pregnancy outcomes, which raises great concerns about the adverse effect of AMA on the long‐term health of offspring. Specific acquired characteristics of parents can be passed on to descendants through certain molecular mechanisms, yet the underlying connection between AMA‐related alterations in parents and that in offspring remains largely uncharted.

**Methods:**

We profiled the DNA methylomes of paired parental peripheral bloods and cord bloods from 20 nuclear families, including 10 AMA and 10 Young, and additional transcriptomes of 10 paired maternal peripheral bloods and cord bloods.

**Results:**

We revealed that AMA induced aging‐like changes in DNA methylome and gene expression in both parents and offspring. The expression changes in several genes, such as *SLC28A3*, were highly relevant to the disorder in DNA methylation. In addition, AMA‐related differentially methylated regions (DMRs) identified in mother and offspring groups showed remarkable similarities in both genomic locations and biological functions, mainly involving neuron differentiation, metabolism, and histone modification pathways. AMA‐related differentially expressed genes (DEGs) shared by mother and offspring groups were highly enriched in the processes of immune cell activation and mitotic nuclear division. We further uncovered developmental‐dependent dynamics for the DNA methylation of intergenerationally correlated DMRs during pre‐implantation embryonic development, as well as diverse gene expression patterns during gametogenesis and early embryonic development for those common AMA‐related DEGs presenting intergenerational correlation, such as *CD24*. Moreover, some intergenerational DEGs, typified by *HTRA3*, also showed the same significant alterations in AMA MII oocyte or blastocyst.

**Conclusions:**

Our results reveal potential intergenerational inheritance of both AMA‐related DNA methylome and transcriptome and provide new insights to understand health problems in AMA offspring.

## Graphical abstract

1. A series of advanced maternal age (AMA)‐related changes are identified in the parental and offspring's DNA methylome, as well as the maternal and offspring's transcriptome.
2. Specific DNA methylome and transcriptional changes present intergenerationally correlation.
3. Part of the AMA‐related differentially expressed genes shared by mother and offspring groups, such as *HTRA3*, were already significantly changed in MII oocyte or blastocyst.

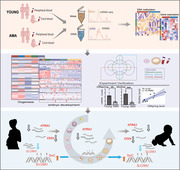

## 1 INTRODUCTION

Advanced maternal age (AMA) is defined as a maternal age of 35 years or older at the time of delivery.^1^ During the past three decades, the percentage of AMA mothers has rapidly increased in many developed and high‐income countries, reaching 23% in the United States in 2014 and as high as 33.4% in Korea in 2019.^1^, ^2^ However, aging, as an inevitable biological progress, comes with the accumulation of organ functional decline and cell damage, such as cardiovascular homeostasis disruption, systemic inflammation, mitochondrial dysfunction and so on.^3^, ^4^ Human plasma proteome research further revealed nonlinear changes during the aging process, with one noticeable crest of protein expression changes around age 34.^5^ In fact, AMA is well recognized as a major risk factor for various pregnancy complications and adverse pregnancy outcomes including preeclampsia, gestational diabetes mellitus, miscarriage and preterm delivery.^6^, ^7^

Numerous studies have indicated that specific parental environmental experiences and physiological changes, such as stress and starvation, could be engraved in nongenetic mechanisms and transmitted to progeny, thereby influencing their phenotypes.^8^ Aging‐related alterations are also reflected by various molecular hallmarks, including genomic instability and increased aberrant transcriptional and DNA methylation. The DNA methylome pattern of a specific set of CpGs is even referred to as the “epigenetic clock” for its accuracy in estimating biological age.^9^ Previous studies have revealed that global epigenetic reprogramming during gametogenesis and embryo development remodel epigenetic patterns.^10^, ^11^ However, 20%–30% of DNA methylation can escape from the first epigenetic reprogramming during preimplantation embryo development, which is regarded as one important mechanism of epigenetic intergenerational inheritance to pass on the specific parental features to the offspring.^12^, ^13^ Only a few parental DNA methylation patterns are able to avoid the much stronger second round of epigenetic reprogramming that occurs during gametogenesis.^11^ This may mediate the inheritance of some special phenotypes over multiple generations, a process defined as the epigenetic transgenerational inheritance.^13^ Consistent with this, previous studies have pointed out that parental age might disturb different characteristics of offspring,^14^ such as the famous “Lansing effect,” which describes the shorter lifespan of the offspring of older parents,^15^ and abnormal offspring behavioural phenotypes associated with DNA methylation abnormalities in sperm from older fathers.^16^ In particular, women over the age of 35 generally suffer from a dramatic decrease in fecundity.^17^ Apart from a well‐documented increase in aneuploid,^18^ AMA has been reported to be accompanied by dysregulation of both the DNA methylome and transcriptome in oocytes,^19^, ^20^, ^21^, ^22^, ^23^, ^24^, ^25^ as well as an overall decrease in gene expression in blastocysts.^26^ Meanwhile, researchers have also claimed that offspring delivered by AMA mothers would face higher risks of autism and impaired cardiometabolic health.^27^, ^28^

Unexpectedly, studies on the epigenomic and transcriptomic impacts of AMA on either pregnant women or offspring are very scarce,^29^, ^30^ and discussions about the concrete relationships of molecular alterations in parents and offspring are even less well studied. In fact, to date, only three research groups have identified CpG sites that were correlated with maternal age, in neonatal heel blood, cord blood and the peripheral blood of adult daughters, separately.^31^, ^32^, ^33^ Hereby, we performed integrated analysis of the transcriptome and DNA methylome of nuclear families and revealed the potential phenomenon of intergenerational inheritance of AMA‐related alterations in both the DNA methylome and transcriptome. We also confirmed that specific abnormal changes in offspring might originate from the disturbance of oocytes or embryos by AMA. Hence, our data provide valuable resources and new insights for understanding the molecular mechanisms underlying the influence of AMA and interpreting the health problems in the later life of AMA offspring.

## 2 RESULTS

### 2.1 AMA‐related abnormal changes in the parental DNA methylome

**FIGURE 1:**
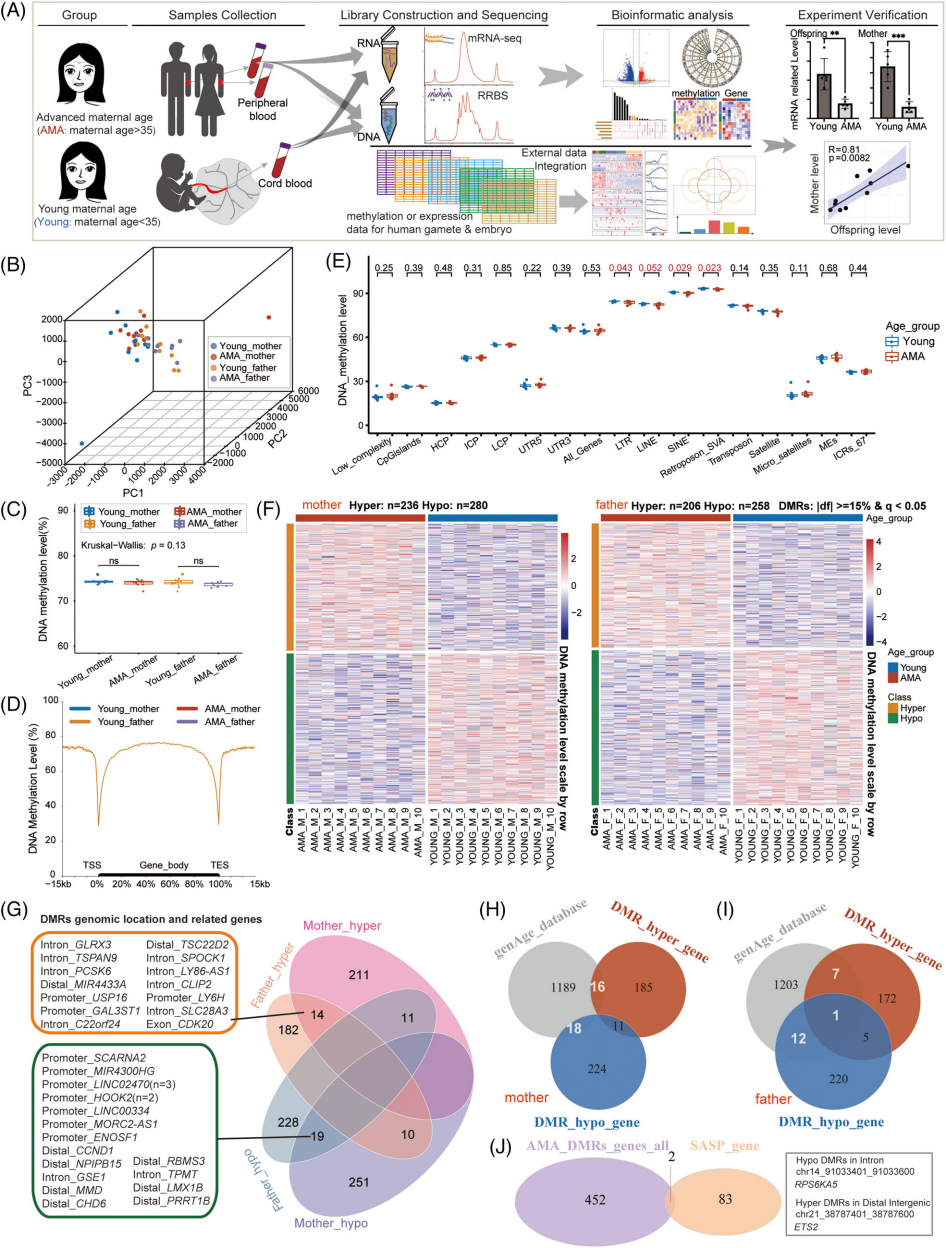
Distinct advanced maternal age (AMA)‐related DNA methylation changes in the mother and father groups. (A) Flowchart of the experiment and data analysis. (B) Three‐dimensional scatter plot showing the distribution of parental samples in the four groups (AMA‐mother, Young‐mother, AMA‐father, Young‐father). The first three principal components from principal components analysis (PCA) based on the 200 bp tiles DNA methylation pattern (*n* = 701937) were used; sample size: *n* = 40. (C) Box plot showing the distribution of DNA methylation level in four groups. Each dot represents the average DNA methylation level of each sample; the *p* value between AMA and Young groups was determined by Wilcoxon rank‐sum test. (ns: *p* > .05); the *p* value for the comparison among multiple groups was determined by Kruskal–Wallis test. (D) Mean DNA methylation levels along with the gene bodies, and 15 kilobases (kb) upstream of the transcription start site (TSS) and 15 kb downstream of the transcription end site (TES) of all genes. (E) Box plot presenting the distribution of the average DNA methylation level of specific genome elements in the AMA‐mother group (red) and Young‐mother group (blue); each dot represents the average DNA methylation level for each sample; the *p* value between the AMA and Young groups was determined by the Wilcoxon rank‐sum test. (F) Heatmap showing the DNA methylation of AMA‐related differentially methylated regions (DMR) in the mother group (left) and father group (right). Blue bars represent the Young group, and red bars represent the AMA group. Orange bars represent hyper DMRs, while green bars represent hypo DMRs. (G) Venn diagram showing the numbers of overlapping and nonoverlapped DMRs among the four groups; the corresponding relative genic location and nearby gene for each DMR in the targeted categories are presented on the left. Distal stands for distal intergenic. (H, I) Venn diagram showing the number of intersections between genes provided by the genAge database and genes near AMA‐DMRs in either the mother groups (H) or the father group (I). (J) Venn diagram showing the number of intersections between genes near AMA‐DMRs and senescence‐associated secretory phenotype genes in the mother groups. The relative genic locations and concrete genomic coordinates for the corresponding DMRs of overlapping genes are presented on the right (ns: *p* > .05, ∗*p* < .05; ∗∗*p* < .01, ∗∗∗*p* < .001).

To investigate the influence of AMA on the DNA methylome of parents and offspring, we applied reduced‐representation bisulphite sequencing (RRBS) to umbilical cord blood (UCB) samples and corresponding parental peripheral blood (PPB) samples from nuclear families. Those samples were further classified into AMA and Young groups based on maternal age (AMA: maternal age of 36–43 years; Young: maternal age of 22–29 years) (Figure 1A). There were no significant differences in maternal BMI or any clinical features of offspring between the AMA and Young groups (see Table S1 for more details). The genome‐wide copy number variations (CNVs) were normal in all samples (Figure S1A). The genome was then split into 200 bp bins, and only bins containing more than three CpG sites and conserved in 80% of the samples in each group were used for downstream analysis (Figure S1B).

Principal component analysis (PCA) and hierarchical clustering analysis showed no obvious separation of the AMA and Young groups in either maternal or paternal samples (Figure 1B and Figure S2A,B). The global patterns of DNA methylation levels were also similar among samples (Figure S2C,D). The mean value was approximately 75% in each group, and it was slightly lower in the AMA group than in the Young group for both maternal and paternal samples, but the difference did not reach statistical significance (Figure 1C,D). Meanwhile, the overall DNA methylation level around the gene body was similar among the four groups (Figure 1D). Interestingly, it was notable that the average methylation levels of all four kinds of retrotransposons (including LTR, LINE, SINE and SVA) were significantly lower in AMA‐mother than in Young‐mother, while no differences were observed in the father group (Figure 1E and Figure S2F).

We next performed intergroup comparisons to elucidate the definitive influence of AMA, and identified 516 and 464 AMA‐related differentially methylated regions (DMRs) in the mother and father groups, respectively (|df|> = 15% and *q* value < .05; for mothers: hyper DMRs (45.7%), *n* = 236; hypo DMRs (54.3%), *n* = 280; for fathers: hyper DMRs (44.4%), *n* = 206; hypo DMRs (55.6%), *n* = 258) (Figure 1F and Figure S3A–F; see Table S3 for more details). These DMRs were broadly distributed on the genome scale and primarily located outside of the promoter regions (Figure S3J,K). Further functional genomic annotation revealed that a large percentage of DMRs were located in SINEs (37.4%–43.4%), LTRs (11.4%–19.9%), LINEs (10.7%–16.1%), as well as CpG islands (4.7%–7.8%) (Figure S4A). However, mother‐DMRs and father‐DMRs were quite distinct in terms of their exact genomic positions, with only 14 hyper‐DMRs and 19 hypo‐DMRs in common (Figure 1G). Searching for the nearest genes of DMRs in both longevity and aging databases^34^, ^35^ revealed that 44 genes in the mother group and 20 genes in the father group had been reported to be associated with aging (Figure 1H,I). The nearest genes of two mother‐DMRs, *RPS6KA5* and *ETS2*, were previously defined as senescence‐associated secretory phenotype (SASP) genes^36^ (Figure 1J), while no overlap existed between the nearest genes of father‐DMRs and SASP genes (Figure 1J). Gene Ontology (GO) enrichment analysis revealed that the nearest genes of mother‐DMRs were enriched in processes involving organ morphogenesis, histone modification, phospholipid metabolism, and leukocyte differentiation. However, the nearest genes of father‐DMRs were highly enriched in wound healing, cell‐substrate adhesion, and calcium ion transmembrane transport (Figure S4B). Both mother‐DMRs and father‐DMRs were enriched in the processes associated with neuron differentiation (nearest genes: *CCND1*, *SPOCK1*), protein homotetramerization (nearest gene: *USP16*) and glycosylation (nearest genes: *GAL3ST1*) (Figure 1G and Figure S4B; see Table S4 for details). Together, the above results indicated that aging‐related alterations in the parental DNA methylomes were present in AMA pregnancy and were more conspicuous in the maternal part.

### 2.2 AMA induced DNA methylation changes in offspring

**FIGURE 2:**
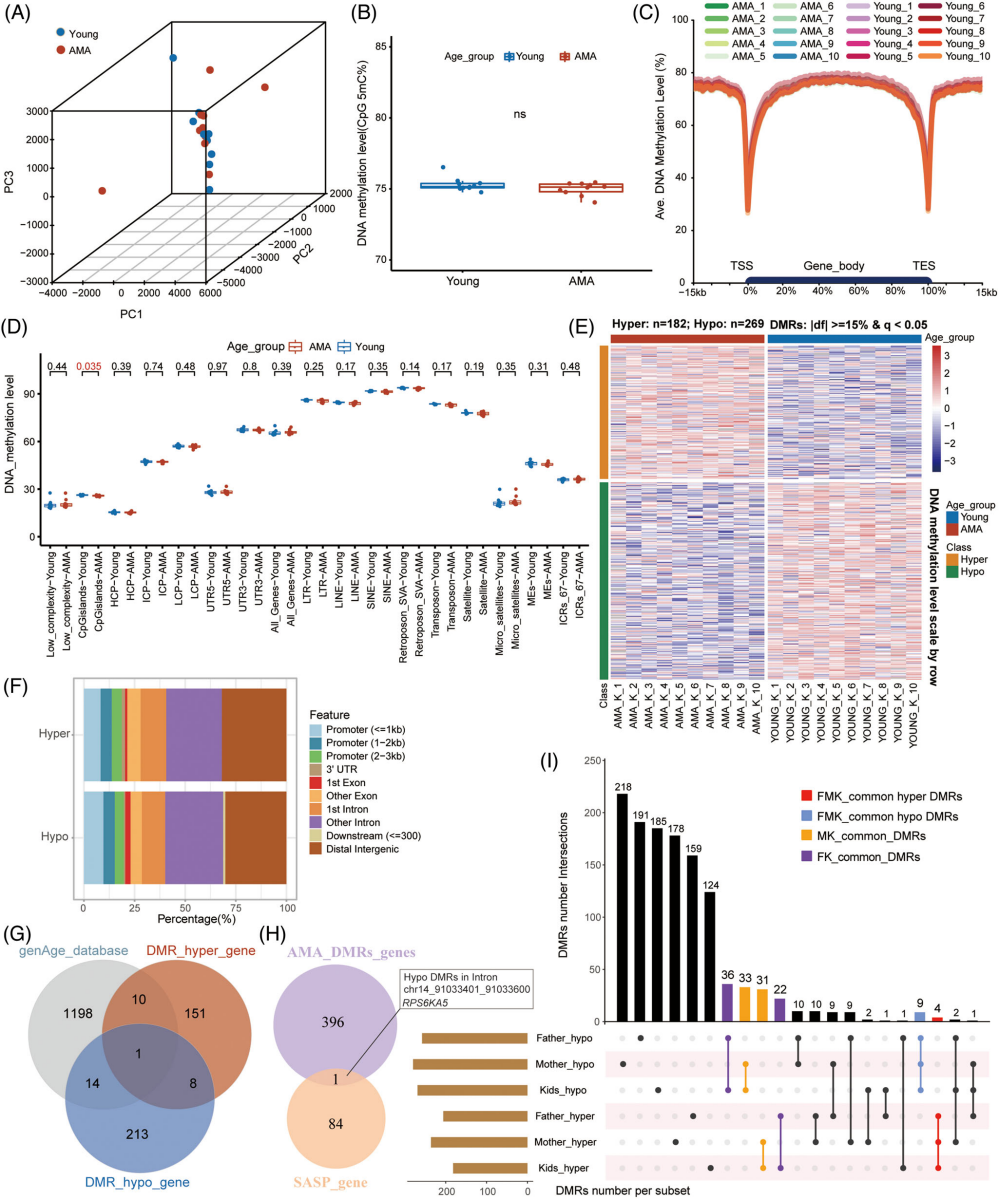
Advanced maternal age (AMA)‐related DNA methylation changes in offspring. (A) Three‐dimensional scatter plot showing the distribution of neonatal samples in the AMA and Young groups (sample size: *n* = 20); the first three principal components of PCA based on the 200 bp tiles DNA methylation pattern (*n* = 701937) were used. (B) Box plot showing the distribution of the mean DNA methylation level in the AMA and Young groups; each dot represents the average DNA methylation level of the corresponding sample; the *p* value between the AMA and Young groups was examined by the Wilcoxon rank‐sum test. (ns: *p* > .05). (C) Average DNA methylation levels of the gene bodies, and 15 kb upstream of the TSS and 15 kb downstream of the TES of all genes for each sample. (D) Box plot showing the distribution of the average DNA methylation level of specific genome elements as mentioned in Figure 1E in the AMA (red) and Young groups (blue); each dot represents the average DNA methylation level for each sample; the *p* value between the AMA and Young groups was determined by the Wilcoxon rank‐sum test. (E) Heatmap showing the DNA methylation level in the AMA‐related differentially methylated regions (DMRs) of the AMA offspring group and Young offspring group. Blue bars represent the Young group, and red bars represent the AMA group. The orange bars represent the hyper DMRs, while the green bars represent the hypo DMRs. (F) Column graph showing the proportion of AMA‐DMRs in different genomic features. (G) Venn diagram showing the intersection of genes provided by the genAge database and genes near AMA‐DMRs. (H) Venn diagram showing the intersection of genes near AMA‐DMRs and senescence‐associated secretory phenotype genes. The relative genic locations and concrete genomic coordinates for the corresponding DMRs of overlapping genes are presented on the right. (I) UpSet plot revealing the relationship among six lists of AMA‐DMRs in father groups, mother groups and offspring groups. Bar plots on the left and top represent the number of DMRs in the corresponding group. MK‐common DMRs are DMRs identified as common between the mother and offspring groups; FK‐common DMRs are DMRs identified as common between the father and offspring groups; FMK‐common are DMRs identified as common among the mother, father and offspring groups.

Then, we analysed the DNA methylomes of UCB samples to evaluate the epigenetic changes in AMA offspring. Similar to what was observed in PPB, PCA and hierarchical clustering analyses showed no obvious separation of the AMA and Young groups (Figure 2A and Figure S2A). The mean value showed no significant intergroup difference (Young: 75.31%; AMA: 74.99%; *p* value = .44; Figure 2B), and the average DNA methylation pattern on the genome‐wide scale or specifically around gene bodies was comparable between the AMA and Young groups. (Figure 2C and Figure S2C,D,G). Nevertheless, the average DNA methylation levels of CpG islands significantly decreased in the AMA group (Young: 26.52%; AMA: 26.50%; *p* value = .035; Figure 2D). Intergroup comparison analysis further identified 182 hyper DMRs and 269 hypo DMRs (|df|> = 15% and *q* value <.05) (Figure 2E, Figure S3G–I), which were broadly distributed on all chromosomes and mainly in non‐promoter regions (Figure 2F and Figure S3J). Once again, we observed a larger percentage of hypo DMRs than hyper DMRs (40.4% vs. 59.6%, Figure S3H) and a high proportion of hyper/hypo DMRs located in SINE (36.8% and 43.4%), LTR (11.5% and 19.2%), LINE (15.2% and 18.1%) (Figure S4A), as described for the parental sample.

Among the nearest genes of offspring‐DMRs, 25 genes were listed in the longevity or aging databases (Figure 2G). Remarkably, the SASP gene *RPS6KA5* was also observed among them, with a nearby hypo DMR located in the intron 1 region (chr14: 91033401–91033600) (Figure 2H). As shown in Figure 1J, the same region was also identified as AMA‐related hypo DMR in the mother group. GO analysis suggested that disturbance of those DMRs might influence various processes associated with vesicle‐mediated transport, autophagy, electron transport chain, eye development, and so on (Figure S4C). Importantly, quite a few GO terms were also found in the enrichment analysis of the mother group, including processes involving neuron differentiation, regulation of GTPase signal transduction, metabolism, and histone modification (Figure S4B,C). Integrated analysis between parental DMRs and offspring‐DMRs further uncovered overlapping common DMRs that shared the same trend between offspring and parents (MK_common DMRs: *n* = 64; FK_common DMRs: *n* = 58, FMK_common DMRs: *n* = 13) (Figure 2I). Taken together, our findings indicated that AMA would also disturb the DNA methylome of the offspring. These changes bore many remarkable similarities to those in the maternal DNA methylome.

### 2.3 AMA altered the maternal and neonatal transcriptomes

**FIGURE 3:**
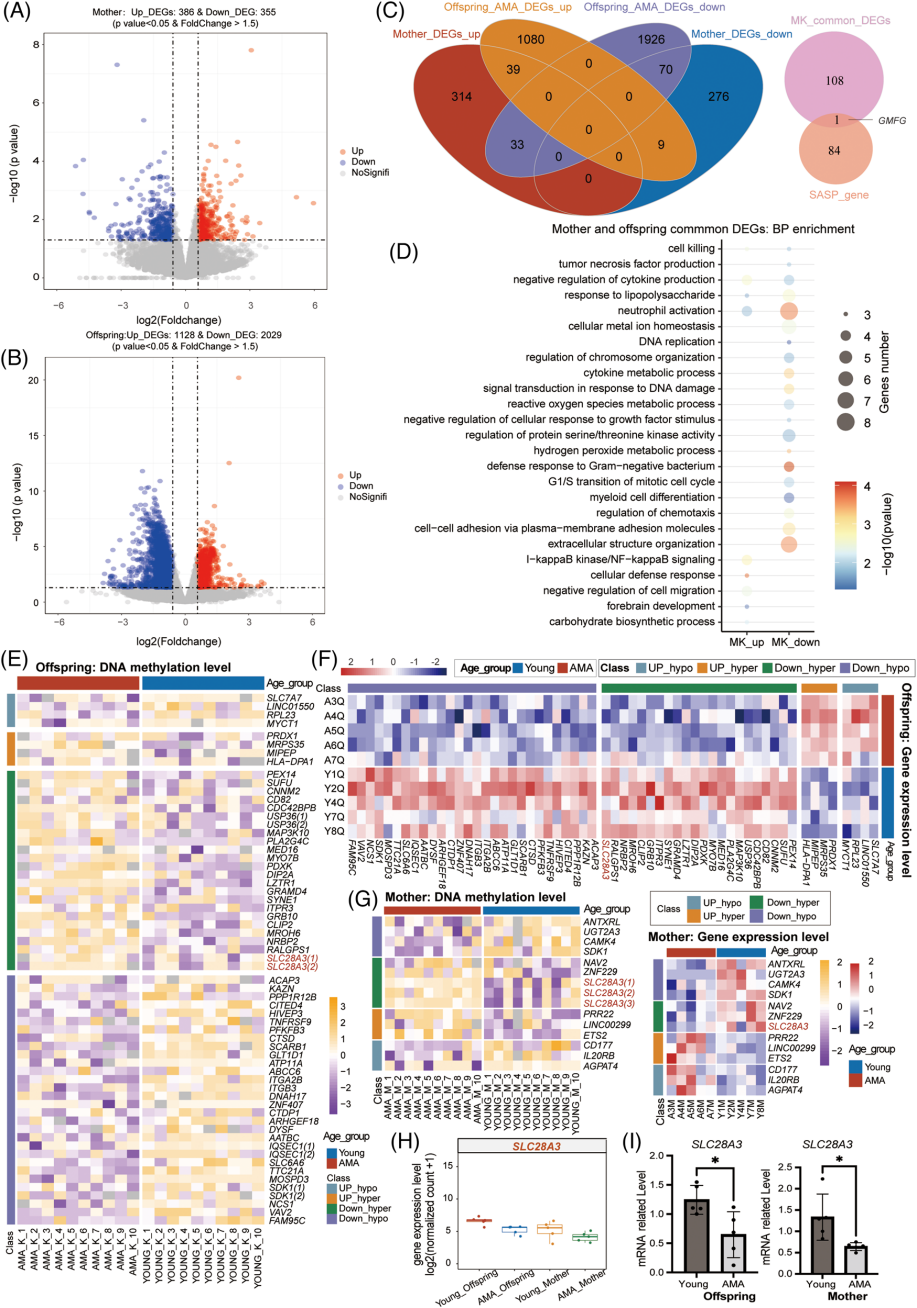
Advanced maternal age (AMA)‐related transcriptional alterations in the mother and offspring. (A, B) Volcano plot simultaneously displaying the *p* value and the fold change in gene expression level in the comparison between the AMA and Young groups for either the mother (A) or offspring (B) groups. Red dots and blue dots represent the upregulated and downregulated DEGs identified between the AMA group and Young group; light grey dots refer to genes with no significant change. (C) Venn diagrams displaying the number of overlapping or no‐overlapping AMA‐DEGs between the mother and offspring groups (left), and the number of intersections between senescence‐associated secretory phenotype genes and common DEGs in mother and offspring groups (right). (D) Bubble chart showing representative Gene Ontology (GO) terms for common AMA‐DEGs mentioned in (C). The gene number in all enrichment terms was not less than three, with a *p* value < .05. The *p* value was measured by hypergeometric test. (E–G) Heatmap showing the DNA methylation level of AMA‐related (differentially methylated regions) DMRs whose nearest genes belonged to AMA‐DEGs in either the offspring group (E) or in the mother group (G, left) or the gene expression level of AMA‐DEGs overlapped with genes near AMA‐DMRs in either the offspring group (F) or in the mother group (G, right). Blue bars indicate the Young group, and red bars indicate the AMA group. Gray and orange bars indicate Up‐DEGs overlapping with genes near hypo DMRs and genes near hyper DMRs, respectively. Green and purple bars indicate Down‐DEGs overlapping with genes near hypo DMRs and genes near hyper DMRs, respectively. (H) Box diagram showing the gene expression level of *SLC28A3* in the four groups (AMA‐Offspring, Young‐Offspring, AMA‐mother, Young‐mother). (I) Column diagram displaying the relative gene expression level of *SLC28A3* determined by qRT‐PCR in the AMA and Young groups for either neonatal samples (left) or maternal samples (right). Each dot represents the relative gene expression level of each sample; error bars refer to the standard deviation. The *p* value between the AMA and Young groups was determined by unpaired *t*‐test (∗*p* < .05).

In addition to examining the DNA methylome, we also performed mRNA‐seq of maternal peripheral blood and UCB samples to explore the influence of AMA on gene expression (Table S1 and Figure S5A). Similar to the observations in the DNA methylome, correlational analysis and PCA based on the transcriptomes of all samples showed obvious separation of the mother and offspring groups but only slight separation between the AMA and Young groups (Figure S5B,C). Intergroup comparison analysis identified 741 and 3157 differentially expressed genes (DEGs) in the mothers and offspring, respectively (Figure 3A,B, Figure S5D,E; *p* < .05 and fold change > = 1.5; for mothers: Up‐regulated DEGs: *n* = 386 and Down‐regulated DEGs: *n* = 355; for offspring: Up‐regulated DEGs: *n* = 1128 and Down‐regulated DEGs: *n* = 2029; see Table S5 for details). A total of 206 DEGs in the offspring group and 43 DEGs in the mother group were recorded in the longevity or aging databases (Figure S5E). GO analysis showed that both offspring‐DEGs and mother‐DEGs were involved in neutrophil activation, mitotic nuclear division, and neuron development. Offspring‐DEGs also focused on those processes related to embryo and placenta development, protein modification, regulation of RNA processing and translation, and mitochondrial function, while mother‐DEGs were highly enriched in mesenchyme development, regulation of inflammatory response, chromosome segregation, and cell−cell adhesion (Figure S5F, see Table S6 for details).

A total of 109 common AMA‐DEGs shared the same trend between the mother and offspring groups (Figure 3C, common Up‐regulated DEGs: *n* = 39 and common down‐regulated DEGs: *n* = 70). *GMFG*, as a SASP gene within this set, was upregulated in the AMA group (Figure 3C). GO enrichment analysis suggested that these DEGs might be involved in the processes of cytokine production, cell mitosis, protein modification, neutrophil activation, defence response to gram−negative bacterium and the NF–KB signalling pathway (Figure 3D; see Table S7 for details). Overall, the above results revealed that the fluctuation in the transcriptome reflected the aging‐like effect of AMA on both offspring and mothers, which shared many similarities with DMRs in related biological processes. The results also presented considerable similarities in the transcriptional alterations between the mother and offspring.

We then performed an integrated analysis between DEGs and DMRs for either mother or offspring to identify DEGs potentially rooted in the DNA methylation change of nearby DMRs (offspring‐DEGs, *n* = 58; mother‐DEGs, *n* = 13) (Figure 3E–G). Specifically, *SLC28A3*, a gene encoding a nucleoside transporter, was significantly downregulated in both the offspring and mothers of the AMA group, along with an increased DNA methylation level in DMRs located in intron 1(Figure 3E–H). The reduced expression of *SLC28A3* was further validated by real‐time fluorescence quantitative polymerase chain reaction (qRT‐PCR) (Figure 3I). This result suggested the highly interconnected changes in the transcriptome and DNA methylome in the AMA group.

### 2.4 Specific alterations in AMA offspring presented intergenerational correlation

**FIGURE 4:**
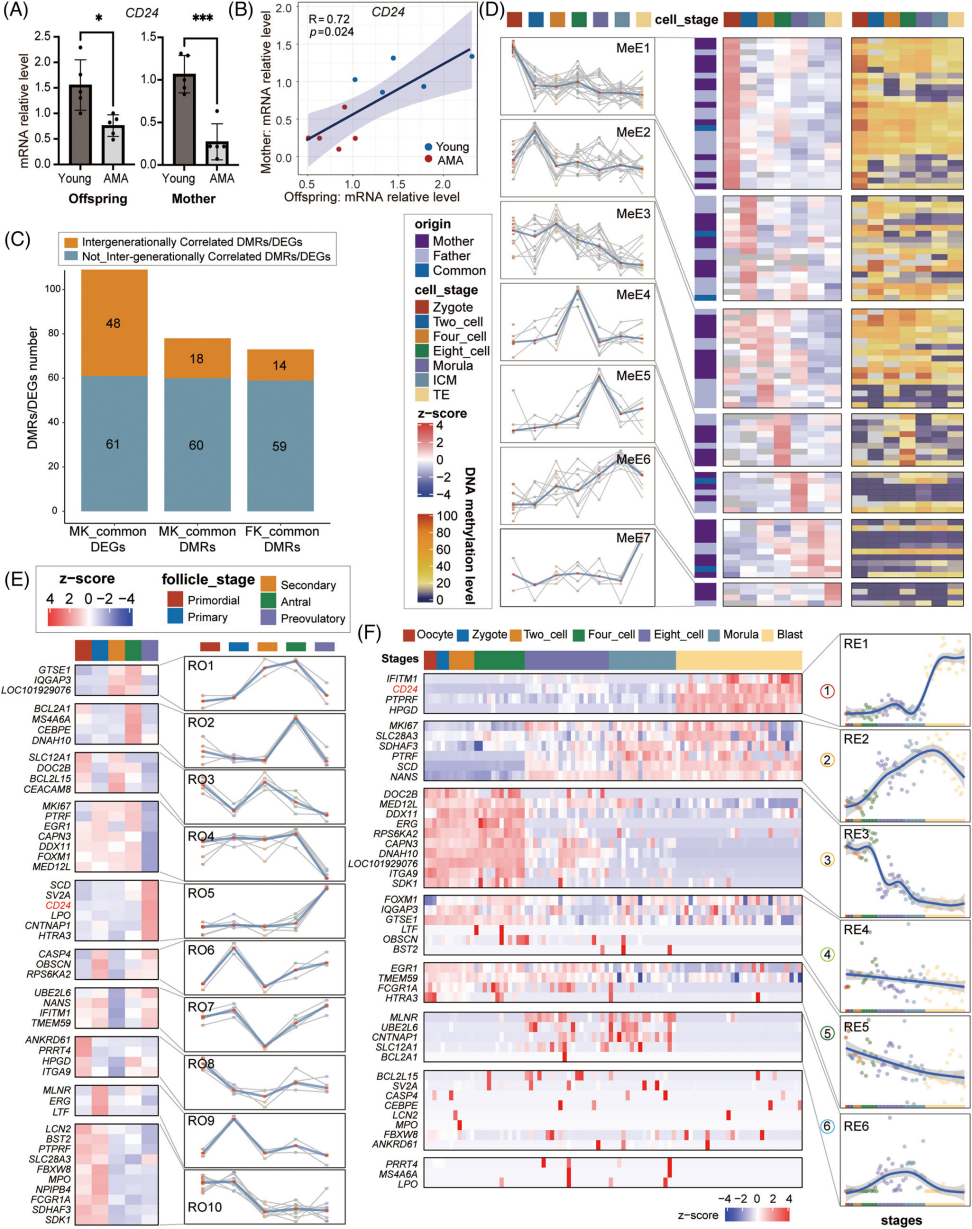
Intergenerational correlation of advanced maternal age (AMA)‐related changes in either the DNA methylome or transcriptome. (A) Column diagram displaying the relative gene expression levels of *CD24* determined by qRT‐PCR in the AMA and Young groups for either neonatal samples or maternal samples. Each dot represents the relative gene expression level of each sample. Error bars refer to the standard deviation. The *p* value between AMA and Young groups was calculated by unpaired t test. (∗*p* < .05; ∗∗*p* < .01; ∗∗∗*p* < .001). (B) Scatter diagram showing the relative gene expression level determined by qRT‐PCR for *CD24* in paired maternal and neonatal samples. The blue straight line is the fitted linear regression line. Each dot represents a family. AMA families are shown in red, and Young families are in blue. The correlation coefficient and *p* value between the mother and offspring groups were calculated by Spearman‐based correlation tests. The shadow indicates the 0.95 confidence level interval around the fitted linear regression line; *n* = 10. (C) Bar graph showing the number of intergenerational and no‐intergenerational correlated differentially methylated regions (DMRs) in either MK‐common DMRs or FK‐common DMRs identified in Figure 2I, as well as the number of intergenerational and no‐intergenerational correlated DEGs in MK‐common DEGs as mentioned in Figure 3C. (D) Heatmap (right) showing the raw average DNA methylation level in different stages of early embryo development for each intergenerationally correlated DMR. Heatmap (left) and line graph showing the scaled average DNA methylation level in different stages of early embryo development for each intergenerationally correlated DMRs. Each DMR was expanded to an additional 150 bp upstream and downstream. Only expanded DMRs covering more than three CpGs in DNA methylome data of early embryo development were analysed. Red dot in each stage refer to the median value of the scaled DNA methylation level of different DMRs. K‐means clustering based on the methylation dynamic pattern was performed to cluster those DMRs. (E) Heatmap and line graph showing the scaled average gene expression level in different stages of human oogenesis for each detected intergenerationally correlated DEGs. Red dot in each stage referred to the median value of scaled gene expression level of different DEGs. K‐means clustering based on the expression pattern was applied to classify those genes. (F) Heatmap showing the scaled gene expression level in each cell during early embryo development for each detected intergenerationally correlated DEG. K‐means clustering based on the expression pattern is applied to classify those genes. Curve chart in the right showing the loess‐smoothed row‐scaled expression dynamics pattern for genes in selected six different clusters. The shadow denotes the 0.95 confidence level interval around the fitting curves. For specific cluster, each point refers to the median of row‐scaled expression value of DEGs in each cell.

Although common changes between parents and offspring have been observed in both the transcriptome and DNA methylome, how close the connection between the changes in the offspring and parents is remains uncertain. In fact, the *CD24* (CD24 molecule), one of the common DEGs and encoding a glycosylphosphatidylinositol‐linked cell surface protein tightly correlated with cell pluripotency,^37^ was not only significantly downregulated in both AMA groups, but also showed strong linear corrections between paired mother and offspring (Figure S8A), which were validated using qRT‐PCR, too (Figure 4A,B). This observation hinted that part of alterations in offspring might be directly correlated with the parental changes induced by AMA. Thus, benefiting from the definite parentage in our cohort, we calculated the Spearman correlation coefficients between parents and offspring for both offspring‐DMRs and offspring‐DEGs to identify the intergenerationally correlated DMRs (*R* > = 0.600 and *p* value < 0.05; Figures S6 and S7) and DEGs (*R* > = 0.600 and *p* value < .05; Figure S8A). Among these, 18 and 14 DMRs, respectively belonged to common mother‐offspring and father‐offspring DMRs shown in Figure 2I (23% and 19%), while 48 DEGs were previously identified as common DEGs showed in Figure 3C (44%; Up‐regulated DEGs: *n* = 11; Down‐regulated DEGs: *n* = 37; Figure 4C). This result suggested that a considerable proportion of AMA‐related DMRs and DEGs observed in offspring might be directly inherited from parents.

The parental characters were passed on to offspring mainly through the gamete and embryo, and previous studies have outlined highly diverse molecular dynamics for different genomic elements and various genes during gametogenesis and pre‐implantation development.^12^, ^38^, ^39^, ^40^ To investigate the DNA methylation pattern of those intergenerationally correlated DMRs during the epigenetic reprogramming process, we here reanalysed the published single‐cell chromatin overall omic‐scale landscape sequencing (scCOOL‐seq) data of human pre‐implantation embryonic development^41^ and depicted the dynamic DNA methylome patterns for 91 intergenerationally correlated DMRs detected in the data (Figure 4D and Table S8). Those DMRs were further classified into seven clusters using hierarchical clustering, and the dynamic pattern in each cluster showed strong development‐stage specificity (MeE1‐MeE7, Figure 4D). It indicated that DNA methylation patterns of those DMRs were strictly regulated by demethylation and remethylation mechanism during embryonic development, rather than just keep out of the epigenetic reprogramming process.

Meanwhile, we also downloaded single‐cell transcriptional data of human oogenesis and pre‐implantation embryos to profile the expression dynamics of 48 intergenerationally correlated common DEGs.^38^, ^40^ Among the 10 patterns defined in follicle generation, genes with the RO2 pattern drastically increased from secondary follicle to antral follicle, a phenomenon that might play important role in follicular lumen formation. Genes with the RO4 and RO5 patterns showed the opposite pattern and striking changes in the preovulatory stage. In particular, Suzhen Yuan and his colleagues found that the upregulation of EGR1 (RO4 pattern) participated in granulosa cell apoptosis and follicle atresia during ovarian aging.^42^ In addition, RO3, RO6, and RO9 patterns presented drastic fluctuations in gene expression from the primordial follicle stage to the primary follicle stage (Figure 4E). Six distinct clusters of gene expression patterns were identified in preimplantation embryos (Figure 4F). The dramatical increase in expression after the morula stage implied that genes with the RE1 pattern might be associated with the cell differentiation of blastocyst. The specific high expression after the 4‐cell stage evinced the character of zygotic genes after major ZGA for genes with the RE2 and RE6 patterns. In contrast that of RE2 gene, the high expression levels of genes with the RE3 pattern decreased sharply after the 4‐cell stage, implying that those genes might be potential maternal‐effect genes that were largely degraded during the maternal‐to‐zygotic transition (MZT). It is worth noting that the expression of *CD24* both increased sharply during the transition from the antral follicle to the preovulatory follicle during folliculogenesis, echoing the early report about its important role in the regulation of ovulation.^43^ During the development of early embryos, the expression of *CD24* was increased in the periods of ZGA and the transition from morula to blastocyst, (Figure 4F), consistent with its high expression in the following villus trophoblast cell and the key roles in mediating immune tolerance at the foetal‐maternal interface.^44^ Together, these results revealed that the DNA methylation patterns of intergenerationally correlated DMRs and the expression patterns of those intergenerationally correlated DEGs varied during oocyte maturation and preimplantation embryonic development, and suggested the potential impact of AMA on multiple key processes in these two periods.

**FIGURE 5:**
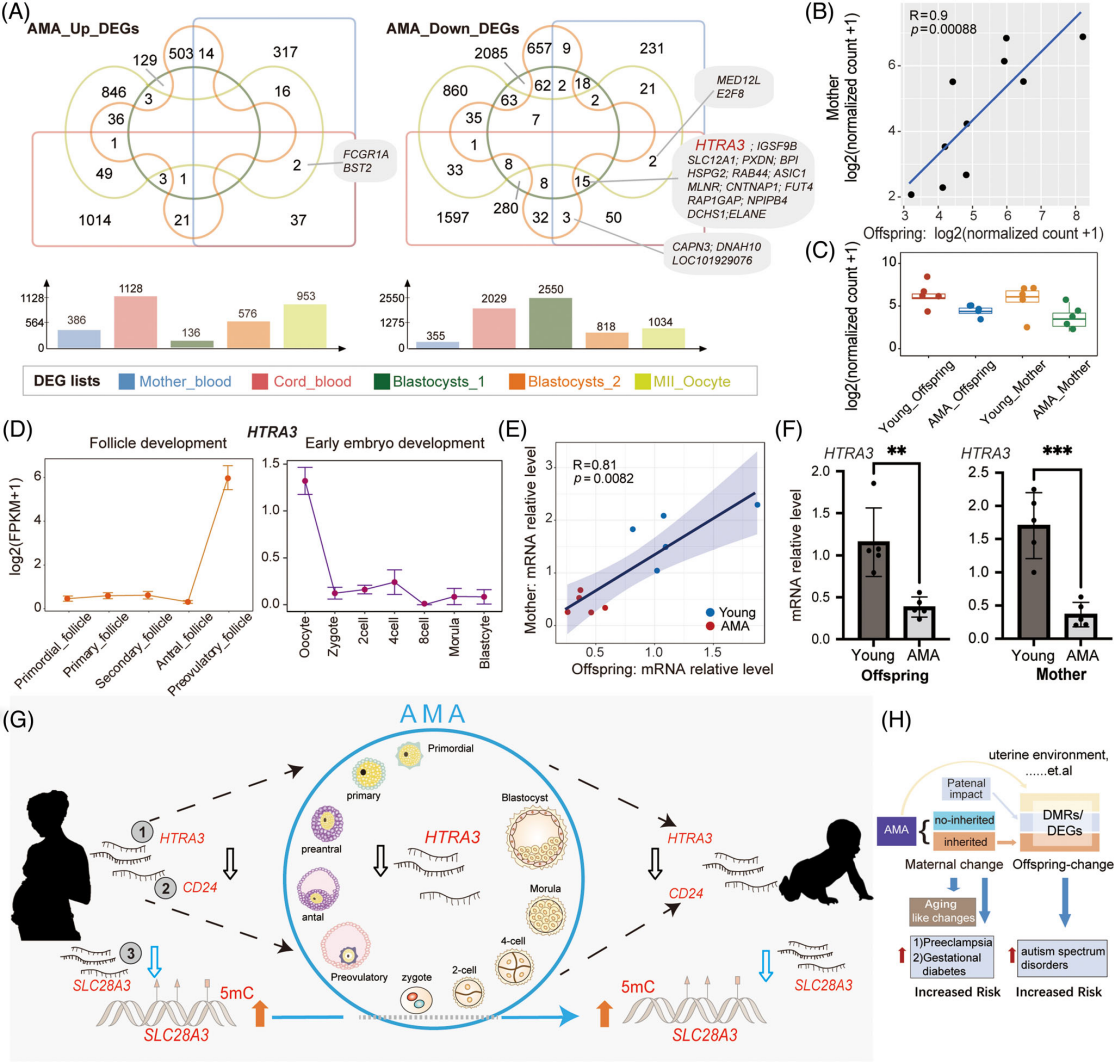
Interrelationship among advanced maternal age (AMA)‐related transcriptional alterations in the mother, offspring, MII oocyte and blastocyst. (A) Venn diagrams displaying the number of intersections for five lists of AMA‐related DEGs from our maternal blood data, cord blood data, one published MII oocyte data and two published cry blastocyst data. Bar graph showing the total number of DEGs in each dataset. The top panel shows upregulated DEGs, while the bottom panel shows downregulated DEGs. (B) Scatter diagram showing the gene expression levels of *HTRA3* in paired maternal and neonatal samples. The blue straight line refers to the fitted linear regression line. The correlation coefficient and *p* value between the mother and offspring groups were calculated by Spearman‐based correlation tests. (C) Box diagram showing the gene expression of *HTRA3* in four groups (AMA‐Offspring, Young‐Offspring, AMA‐Mother and Young‐Mother) (D) Line diagram showing the gene expression dynamics of *HTRA3* during oogenesis and early embryo development. (E) Scatter diagram showing the relative gene expression level determined by qRT‐PCR for *HTRA3* in paired maternal and neonatal samples. The blue straight line is the fitted linear regression line. Each dot represents a family. AMA families are shown in red, and Young families are in blue. The correlation coefficient and *p* value between the mother and offspring groups were calculated by Spearman‐based correlation tests. The shadow indicates the 0.95 confidence level interval around the fitted linear regression line; *n* = 10. (F) Column diagram displaying the relative gene expression levels of *HTRA3* determined by qRT‐PCR in the AMA and Young groups for either neonatal samples or maternal samples. Each dot represents the relative gene expression level of each sample. Error bars refer to the standard deviation. The *p* value between AMA and Young groups was calculated by unpaired *t*‐test. (∗*p* < .05; ∗∗*p* < .01; ∗∗∗*p* < .001). (G) A schematic illustration showing the close connection between mother and offspring for the AMA‐related alterations in either transcriptome or DNA methylome. The AMA‐related alteration in three genes:*SLC28A3*, *CD24* and *HTRA3* represents three typical patterns for intergenerationally correlated AMA‐DEGs mentioned in our study, respectively. AMA's impact on pregnant women will induce abnormal fluctuations in specific gene expression (such as *HTRA3*) in MII oocytes or preimplantation embryos, thus contributing to the similar changes observed in offspring. (H) A schematic diagram showing the interrelationship between AMA, AMA‐related maternal molecular change, AMA‐related offspring molecular change, and disease risk. AMA induced adverse changes on the mother and resulted in the increased risk of pregnant complications. The inheritable part of maternal changes, together with paternal impact and other external factors, lead to abnormal molecular changes in offspring and further influence their long‐term health.

### 2.5 AMA‐related transcriptional alterations existed in AMA oocyte and blastocyst

Several previous studies have discussed the influence of AMA on human MII oocytes or embryos after cryopreservation.^45^, ^46^, ^47^ We then compared offspring‐DEGs and mother‐DEGs with two published blastocyst‐DEG lists^26^, ^46^ and one MII oocyte‐DEG list,^47^ and revealed a series of overlapping DEGs (Figure 5A, see Table S9 for details). Among common AMA‐DEGs between mother and offspring groups, two upregulated genes and two downregulated genes were observed in the oocyte, while 18 genes were downregulated in the blastocyst. Meanwhile, 11 of those 22 genes (*HTRA3*, *FCGR1A*, *BST2*, *MED12L*, *SLC12A1*, *MLNR*, *CNTNAP1*, *NPIPB4*, *CAPN3*, *DNAH10*, and *LOC101929076*) were previously identified as mother‐offspring intergenerationally correlated DEGs (Figure 5A and Figure S8A). In particular, the *HTRA3* (high temperature requirement factor A3), which encodes a serine protease and has been reported to negatively regulate trophoblast invasion,^48^ has a reduced expression in the AMA mother, offspring and blastocyst groups (Figure 5A–C). In folliculogenesis, the expression of *HTRA3* increased sharply during the transition from the antral follicle to the preovulatory follicle, consistent with the previous report about the important role in the regulation of ovulation and luteinization.^49^ In the development of early embryos, the expression of *HTRA3* was downregulated soon after fertilization (Figure 5D). qRT‐PCR further validated the significant downregulation in the AMA group and linear corrections between mother and offspring for *HTRA3* (Figure 5E,F). Among the four DEGs observed in oocyte, *MED12L*, one mother‐offspring intergenerationally correlated DEGs downregulated in the AMA group, showed a trend toward downregulation in both mothers and offspring in qRT‐PCR validation, but statistical significance was reached only in the mother group (Figure S8B,C). In conclusion, these results supported the view that some of the intergenerationally inherited alterations observed in the transcriptome of offspring directly came from the disturbance of AMA in oocytes or preimplantation embryos (Figure 5G,H).

## 3 DISCUSSION

A variety of socioeconomic factors in contemporary society have combined to produce an increase in pregnancies among women of AMA.^50^ In addition to being an independent risk factor for various pregnancy complications, AMA is generally accompanied by greater risks of adverse pregnancy outcomes and adverse effects on the long‐term health of offspring.^27^ Although accumulating evidence has blamed offspring's health issues on pre‐existent AMA‐induced maternal abnormities, the underlying molecular mechanisms for the intergenerational hereditary phenomenon are still largely uncharted. In this study, we systematically profiled the influence of AMA on the DNA methylome and transcriptome of nuclear families and explored the potential origin of offspring changes, which might be inherited through gametes and embryos. We observed a significant reduction in the DNA methylation levels of various retrotransposons in the AMA‐mother group, and on the CpG island in the AMA‐offspring group. Many genes near AMA‐DMRs, as well as AMA‐DEGs, have been reported to be associated with aging. We also identified a series of genes whose expression changes might result from a corresponding alteration in DNA methylation and many common biological processes enriched for both AMA‐DMRs and AMA‐DEGs. This reflected the aging‐like alteration induced by AMA and the consistency of AMA impacts across different omics. Furthermore, we highlighted the similar influence of AMA between parents and offspring, especially between mother and offspring. Combined with published datasets, our analyses further revealed the diverse methylation patterns of intergenerationally correlated DMRs and expression patterns of intergenerationally correlated DEGs presented in human folliculogenesis and preimplantation embryonic development. In particular, some AMA‐DEGs were already significantly changed in MII oocyte or blastocyst, such as *HTRA3*, whose differential expressions was further verified in both the mother and offspring groups. In summary, this research unveiled the intergenerational relatedness of the alterations induced by AMA in both the DNA methylome and transcriptome and supported the claim that the adverse impact of AMA on the mother might interfere with oocytes and early embryos, and thus lead to abnormal changes in offspring.

Specific environmental exposures or experiences and physiological changes, such as starvation, depression and the aging process, could influence the stability of the DNA methylome.^8^ Heijmans and colleagues identified that the DNA methylation changes induced by starvation in the imprinting control region (ICR) near the *IGF2* gene in the mother were associated with the lowered birth weight in offspring.^51^ The accumulation of errors in DNA methylation maintenance during the aging process generally leads to global decreases in DNA methylation.^52^ Puberty and menopause demarcate the beginning and the end of the female reproductive life cycle, while 35 years of age is generally regarded as a turning point for female fecundity, marked by rapid declines of antimullerian hormone (AMH), ovarian reserve and increased risks of adverse pregnancy outcomes.^53^ We observed significant alterations in a range of genes and genomic regions previously reported to be associated with the aging process, longevity and the SASP (such as *RPS6KA5* and *ETS2*) in AMA mothers. Many AMA‐DEGs and AMA‐DMRs were highly enriched in immunity processes and the processes of glycometabolism and cardiovascular function. Higher chronic proinflammatory status has been regarded as one prominent feature of aging.^54^ Meanwhile, aging is well known as a major risk factor for metabolic syndrome, arteriosclerosis and cardiovascular disease.^3^ The above observations together claimed a more universal aging process laying behind the reproductive aging in AMA mothers. Furthermore, this might partly explain the strongly independent association between AMA and the increased risk of gestational diabetes mellitus and preeclampsia.^6^ The DNA methylome of AMA fathers also exhibited a series of alterations, which were enriched in the regulation of neuron projection development and response to wounding, and the number of AMA‐related DMRs identified in fathers was similar to that identified in the mothers. Additional intergenerational contributions from AMA father might partly explain the significantly greater number of offspring‐DEGs compared with mother‐DEGs. A 10‐year birth cohort study in Sweden has suggested a relationship between advanced paternal age and increased incidence of autism in the offspring.^55^ Kaichi Yoshizaki and colleagues further showed that hypomethylation of mouse sperm DNA could alter the expression of genes in REST/NRSF pathway and induce an intergenerational influence on the neurodevelopmental programs in the offspring.^56^ Thus, although the variations in AMA fathers showed less consistency with the aging process, advanced paternal age might also introduce specific intergenerational influence on offspring.

More importantly, we observed significantly downregulated methylation levels at retrotransposons (LTR, LINE, SINE, SVA) in the AMA‐mother group. Retrotransposons make up more than one‐third of the human genome, and show increased expression levels but decreased DNA methylation levels during aging in multiple species.^57^ Our results further demonstrated that AMA might introduce an aging‐like influence into the maternal DNA methylome. Nevertheless, the methylation levels of retrotransposons showed no significant inter‐group difference in fathers, although there was a larger age difference between fathers in the two groups. Recent genome‐wide DNA methylation profiling studies further highlighted the sexual dimorphism in the specific age‐related DNA methylation patterns of CpGs.^58^ Females generally outlive males and have a younger epigenetic age than their male counterparts.^59^ Previous studies discovered that sex hormone fluctuations can respond to changes in epigenetic age within a relatively short time in females.^60^, ^61^ Thus, the much more dramatic changes in sex hormone levels before and after age 35 in females may be the immediate cause of the obvious sexual dimorphism in the methylation changes of retrotransposons. Due to the limited retrotransposon coverage of RRBS on coverage on retrotransposons, it would be better to use WGBS or pyrosequencing to verify the sexual dimorphism of the global DNA methylation change in retrotransposons in the future.

AMA might lead to premature senescence in the term placenta and first trimester villi of either human or mice.^62^ Our study found that 25 genes near AMA‐DMRs and 206 DEGs in AMA‐offspring were associated with aging or longevity. Remarkably, the changes in DNA methylation or gene expression of several aging‐related genes, such as *RPS6KA5* and *GMFG*, in AMA‐offspring coincided with the alterations in AMA‐mothers. Those molecular characteristics in AMA‐offspring indicated that the aging‐like influence of AMA was not limited to extraembryonic tissue. To date, three studies using methylation microarrays have suggested alterations in offspring DNA methylation related to maternal age in childbirth. Markunas et al. claimed that methylation changes in five identified CpGs near *KLHL35* might persist from birth until adulthood.^32^ The 144 CpGs identified by Adkins et al. in offspring and 87 CpGs revealed by Moore et al. in adult daughters were both enriched in the processes associated with neurological regulation and metabolism, which implied that maternal age might affect the metabolism and neurodevelopment in later life of offspring.^31^, ^33^ Our results also presented the DNA methylation changes involved in the regulation of neuron development, Rho protein signal transduction, glycosyl compound metabolic process, hexose metabolic process and catabolic process in AMA‐offspring. This was consistent with the greater risks of neurological and neuropsychiatric diseases observed in AMA offspring in humans,^63^ and the abnormal hippocampal gene expression and impaired learning and memory observed in the AMA offspring of mice model.^29^, ^64^ However, the controversies about the relationship between AMA and the metabolism disorders in the offspring^28^ call for long‐term follow‐up research in larger cohorts. Another notable aspect was the potential disturbance of DNA methylation involved in the respiratory electron transport chain, mitochondrial gene expression and protein targeting to the ER, which were all related to oxidative stress, the key mechanism of endothelial dysfunction and arterial damage, which ultimately increased the risk of vascular disease and arterial stiffness.^65^ In this context, our data supported the points proposed by previous AMA cohorts that AMA offspring are at greater risk of cardiovascular diseases due to oxidative stress.

Previous studies on the relationship between maternal age and the offspring DNA methylome are limited by the lack of maternal methylation data and the potential influence of confounding diseases, such as breast cancer and cleft lip.^31^, ^32^, ^33^ Benefiting from our strict inclusion criteria and the availability of data from both parents and offspring, we were able to directly evaluate the intergenerational inheritance phenomenon of the effects of AMA on either the DNA methylome or transcriptome. Enrichment analysis for 109 common DEGs and 78 common DMRs between the mother and offspring groups suggested their participation in processes involving neuron differentiation, immune and protein modification. Meanwhile, the paired intergenerational analysis further confirmed the high relevance in special genes or genomic regions, and those intergenerationally correlated DEGs and DMRs represented various dynamic patterns during the development of oocyte and early embryo. The stage‐specific DNA methylation patterns for those correlated DMRs supported the view that the drastic de novo DNA methylation happened in every stage, and DNA methylation reprogramming in preimplantation embryo development was shaped by the combination of the global demethylation and local remethylation.^12^ It also implied that those DMRs might play key roles in regulating embryo development, which need further verification. Among those correlated DEGs, genes with the RO4 patterns showed quite striking changes during the preovulatory stage. *EGR1*, a gene belonging to the EGR gene family, has been demonstrated to be induced by LH and encodes transcription factors important for ovulation in mice.^42^ *CAPN3* may have a potential regulatory role in embryonic muscle fibre phenotype and development.^66^ Those genes with RO3, RO6, and RO9 patterns might be involved in primordial follicular activation. The similarity of the dynamic pattern implied that other genes or regions within those clusters might exert similar functions in gametogenesis and embryonic development process, but further investigations are needed.

AMA‐related transcriptional changes in oocytes were significantly enriched in the processes of mitochondrial function, oxidative stress and actin‐binding alteration.^53^ Consistent with this, AMA would also induce a significant global decline in the transcriptome of the blastocyst and repressed the processes related to mitochondrial function and the cell cycle.^26^ Apart from those overlapping AMA‐DEGs (*HTRA3, CNTNAP1, DNAH10, MED12L, BST2*, etc.) among adult blood, cord blood, oocyte and embryo data, AMA‐related changed gene expression associated with the cell cycle and cell division was observed in both the mother and offspring groups. Combined with the potential disturbance of mitochondrial electron leakage and gene expression in offspring, as mentioned earlier, this observation underscores the direct intermediary role of oocyte and embryo as biological stages vulnerable to the influence of AMA. In particular, *HTRA3* and *MED12L* were both decreased intergenerationally correlated AMA‐DEGs, with the former co‐altered in AMA embryos and the latter existing in AMA oocytes. The deletion of *HtrA3* causes the placenta capillaries dysfunction and intra‐uterine growth restriction (IUGR) in mice model.^67^ Thus, the decrease of *HTRA3* in AMA embryos suggested a further investigation of placental abnormalities in AMA pregnancy. *MED12L* (Mediator Complex Subunit 12L) is the homolog of *MED12*, a classical maternal factor that the oocyte‐specific ablation of which in mice would influence embryo development but not disrupt the folliculogenesis and ovulation.^68^ The relatively high expression of *MED12L* was maintained until the 8‐cell stage, and therefore implied that it may also serve as a maternal factor, the abnormal downregulation of which in AMA‐oocyte may also disrupt the following early embryo development. The sharply increased expression of *CD24* in oocyte of preovulatory follicle suggests its important role in oocyte development. Previous study also verified that *CD24* is critical for triggering ovulation.^43^ Although *CD24* did not observe in the AMA‐related DEGs list of MII oocyte provided by Zhang et al.,^47^ our unpublished data do suggest that the expression of *CD24* may also be disturbed in human AMA‐oocyte. Taken together, this evidence indicated that some of the changes in the AMA offspring may be due to the abnormal changes in the gene expression network of oocytes and preimplantation embryos. Our results supported the explanations offered by the developmental origins of health and disease (DOHaD) hypothesis for the later‐life health problems of AMA offspring.^69^

Our research has some limitations. First, although previous research by Adkins et al. found no significant relationship between maternal age and the methylation pattern reflecting blood cell populations, it would be ideal to separate individual cell populations for analysis or perform analysis based on the rapidly developing single‐cell method. Second, studies in larger prospective cohorts will be helpful to validate our observations, to supplement the paired samples of nuclear families with rigorous exclusion criteria applied in our study. Third, only the cord blood was evaluated in our study, and the biopsy tissue from adult offspring and the extraembryonic tissues, such as the villus from the placenta, need to be further investigated for comprehensive and detailed recognition of the influence of AMA across organs and developmental stages. In conclusion, we systematically explored the impact of AMA on the DNA methylome and transcriptome of parents and offspring. AMA might induce intergenerational epigenetic and transcriptional changes involving immune and metabolism pathways, some of which were rooted in oocyte and early embryo, and then passed on to the next generation.

## 4 EXPERIMENTAL PROCEDURES

### 4.1 Ethics

All samples were collected after patients signed informed consent and in accordance with ethical standards. This research was approved by the Reproductive Study Ethics Committee of Peking University Third Hospital (approval number scheme: 2016SZ‐015).

### 4.2 Sample collection and treatment

Thirty nuclear families with foetuses (20 parents‐offspring families and 10 mother‐offspring families) were recruited in this research and were divided into the AMA group and the Young group based on maternal age. The mean age of AMA mothers was 39 ± 1.22 years, while that of young mothers was 29.8 ± 2.59 years. The BMI was not significantly different between AMA and Young mothers. Women with polycystic ovarian syndrome (PCOS), diabetes, hypertension, hyperthyroidism, hypothyroidism, systemic lupus erythematosus, or pregnancy complications such as preeclampsia, gestational diabetes mellitus, severe metabolic syndrome, or intrauterine infection were not eligible. Families with premature babies or infants who suffered from chromosomal abnormalities, any birth defects, or the Apgar score below 7 were also excluded. All women delivered by caesarean section at term (37 to 42 weeks). Approximately 4 ml of UCB and parental peripheral blood samples were collected on the day of delivery and treated within 2 h. Parental peripheral blood samples were collected before delivery in order to avoid any effects of blood transfusion during delivery. Of the 4 ml samples, 1 ml was used for DNA extraction and 1 ml was used for RNA extraction immediately, and the rest was stored at −80°C for backup.

### 4.3 DNA extraction

QIAamp^®^ Blood Mini Kit (Qiagen Cat# 51104) was used to collect genomic DNA (gDNA) from the blood samples following the manufacturer's instructions. The gDNA was evaluated by a NanoDrop 300 ultraviolet spectrophotometer (ALLSHENG#AS‐11020‐00) to ensure an A260/A280 value ranged between 1.8 and 2.0, and stored in −80°C.

### 4.4 RNA extraction

QIAamp RNA Blood Mini Kit (Qiagen Cat# 52304) was used to extract total RNA from the whole blood sample. The RNA was evaluated a NanoDrop 300 ultraviolet spectrophotometer (ALLSHENG#AS‐11020‐00) and Agilent 2100 Bioanalyzer (Agilent# G2939BA). Qualified RNA samples were referred to as samples with an A260/A280 value of approximately 2.0 and an RNA integrity number (RIN) of no less than 7. A total of 500 ng of total RNA was reverse transcribed to obtain cDNA using the PrimeScript^™^ RT reagent Kit (TaKaRa Cat# RR047A). The remaining RNA sample was applied for library construction for mRNA‐seq.

### 4.5 Reduced representation bisulphite sequencing library construction and sequencing

RRBS was performed as follows: The digestion of 3 ng unmethylated lambda DNA (Thermo Scientific, Cat# SD0021) and 500 ng of gDNA in 40 μl mixture systems was performed by adding 5 μl FastDigest MspI and 5 μl 10x FastDigest buffer (Thermo Scientific Cat# FD0544). Then, the end‐repair and adapter ligation of the digested DNA fragments were handled with the NEBNext Ultra DNA Library Prep Kit (NEB Cat# E7370), following the recommended instructions in the manual. Specifically, NEBNext methylated adapter (15 μM; NEB Cat# E7535) was used to prevent the change in adaptor sequence during bisulphite conversion treatment. After the digestion of U bases by 3 μl USER enzyme (NEB Cat# E6610A), size selection of DNA fragments was performed through agarose gel electrophoresis (2% TAE gel) and excision of gel slices containing targeted 200–700 bp DNA, which were extracted by a gel DNA recovery kit (VISTECH Cat# DC2005). Next, a MethylCode bisulphite conversion kit (Thermo Scientific Cat# MECOV‐50) was used for bisulphite conversion according to the manuals. The converted fragments were amplified and tagged with specific barcode sequences by 12 cycles of PCR using Kapa HiFi U+ Master Mix (Kapa Biosystems Cat# KK2801). Finally, the product was purified by two rounds of clean‐up using 0.8X Agencourt AMPure XP Beads (Beckman Cat# A63881), followed by quality testing in a Qubit 3.0 Fluorometer (Thermo Scientific Cat# Q33216). After the detection of exact fragment distributions and molar concentrations with a Fragment Analyzer^™^ Automated CE System (Analysis Kit: Cat# DNF‐474‐0500) and a Library Quant Kit for Illumina (NEB Cat# E7630L), the qualified DNA methylation libraries were sequenced on the Illumina NovaSeq platform with the PE150 strategy.

### 4.6 mRNA‐seq library construction and sequencing

mRNA libraries were generated using the NEBNext^®^ UltraTM RNA Library Prep Kit for Illumina^®^ (NEB Cat# E7530L) according to the manufacturer's recommendations. In short, poly(A) mRNA was isolated from approximately 500 ng total RNA with NEBNext Magnetic Oligo d(T)25 Beads provided in NEBNext Poly(A) mRNA Magnetic Isolation Module (NEB Cat #E7490). After fragmentation, priming, and the subsequent first‐strand and second‐strand cDNA synthesis, double‐stranded cDNA was obtained and purified by 1.8X AMPure XP Beads (Beckman Cat# A63881). Then, the end preparation and adapter ligation of double‐stranded DNA fragments were performed, and the hairpin loop structure within the adaptor was cut by incubating the product with USER Enzyme (NEB Cat# M5505L). Finally, purified adaptor‐ligated DNA fragments were amplified and tagged with specific barcode sequences by PCR. The final mRNA libraries were assessed and sequenced as previously described in the RRBS protocol.

### 4.7 Real‐time quantitative reverse transcription polymerase chain reaction

qRT‐PCR for reversed cDNA was performed with PowerUp^™^ SYBR^™^ Green (Thermo Fisher Cat# A25742) in the QuantStudio 3 Real‐Time PCR system as follows: 95°C for 5 min, followed by 40 cycles of 95°C for 30 s, 60°C 40 s and 72°C 1 min, followed by 72°C 5 min. The delta‐delta‐Ct (^ΔΔ^Ct) algorithm was performed to calculate relative gene expression.  Each experiment was performed three times, and *ACTB* was used as the control. The primers are listed below:

***SCD*** (F: ACGCTTGTGCCCTGGTATTT/R: GCACCACAGCATATCGCAAG)

***CD24*** (F: GCTCCTACCCACGCAGATTTA/R: GACCACGAAGAGACTGGCTG)

***SLC28A3*** (F: TGTCAGCACCTGCGTCAT/R: CCTGCCATTCCACTCCC)

***HTRA3*** (F: CTGTGTTGTTGCTGGGTCAC/R: GTGTTCTGTAGGGCGAAGGG)

***MED12L*** (F: CTCCCTCAGTATCCAGGGCT/R: CTGCTGCAAAGGCATCTGTG)

***ACTB*** (F: CATGTACGTTGCTATCCAGGC/R: CTCCTTAATGTCACGCACGAT)

### 4.8 Data downloading and processing

The single‐cell gene expression matrix for human oocytes during folliculogenesis and pre‐implantation embryo was downloaded from our previously published datasets.^38^, ^40^ The expression level was estimated using the fragments per kilobase million (FPKM). The scCOOL‐seq data of human preimplantation embryos were downloaded from previously published datasets,^41^ and processed as previously described. Briefly, Bismark software (version 0.23.0)^70^ was applied to align qualified reads to the *Homo sapiens* reference genome (human GRCh38/hg38) with the parameter “–paired‐end and –non_directional.” Those unmapped reads were then re‐aligned with single‐end and non‐directional model. After removing the PCR duplicates, the methylation levels of CpG sites were extracted using the function bismark_methylation_extractor" in Bismark software (version 0.23.0) for downstream analysis. The aging and longevity genes list were downloaded from the Aging Atlas^35^ and LongevityMap (Build 3).^34^

### 4.9 Fundamental analysis for RRBS data

All 150 bp bisulphite sequencing paired‐end reads were trimmed to delete adaptors, bases of substandard quality (Q < 20), and the reads shorter than 36 bases using TrimGalore software (https://www.bioinformatics.babraham.ac.uk/projects/trim_galore/; version 0.6.6) and Cutadapt (version 1.18), with the parameters“ –quality 20 –phred33 –stringency 3 –length 36 –rrbs –paired –trim1.” For bisulphite conversion rate evaluation, phage λ genome was used as an extra reference using the function “bismark_genome_preparation” in Bismark software (version 0.23.0).^70^ Bismark with the parameter “bowtie2" was used to map the clean reads to the spiked‐in phage λ genome, and the bisulphite conversion rate was determined by the ratio of the number of unmethylated Cs to the total number of Cs detected. Sixty samples with a bisulphite conversion rate of more than 99% were retained for downstream analysis. Subsequently, Bismark with the parameter “bowtie2” was performed to align clean reads to the *H. sapiens* reference genome (human GRCh38/hg38). Only the uniquely mapping readings with less than 2% mismatch were retained. Then, the number of reads supporting Cs and supporting Ts in each CpG site was counted using the function “bismark_methylation_extractor” in Bismark with the parameter ” –paired‐end –no_overlap." Finally, the coverage files recording the methylation state of CpG sites were inputted into the R package methylKit (version 1.10.0) for further analysis, and only CpG sites on autosomes with more than 5‐fold read coverage were retained. The genome was tiled into consecutive 200 bp windows, and the 200 bp bins covered more than three CpG sites and existed in at least eight samples per group (AMA‐offspring, AMA‐mother, AMA‐father, Young‐offspring, Young‐mother, and Young‐father) were retained for downstream analysis. Detailed information including sequencing depth and bisulphite conversion rate, the number of covered CpG sites and retained bins, is provided in Table S2.

### 4.10 Copy number variation analysis

For each RRBS library, the software “readCounter” in the HMMcopy suite following the R package “HMMcopy” (version 1.26.0) was applied to calculate the CNVs at 1 Mb resolution based on mapped RRBS reads sorted by SAMtools (version 1.3.1). The CNVs were plotted by the R function “points” and “plot.”

### 4.11 Global DNA methylation level estimation and differentially methylated regions identification

The DNA methylation level of any retained 200 bp bin was calculated using the R package methylKit (version 1.10.0)^71^ as the ratio of the total count of Cs and the total count of Cs and Ts bases within that bin. Based on this, the methylation level of each sample was calculated by averaging the DNA methylation levels of all bins. Intergroup comparisons between the AMA and Young groups were also performed using the R package methylKit (version 1.10.0),^71^ and DMRs was referred as 200 bp bins with q‐values no more than .05 and the mean methylation difference thresholds no less than 15%.

### 4.12 DNA methylation pattern around the genic region

The ‐15 kb upstream of the TSS and 15 kb downstream of the transcription end site (TES) of each gene were separately split into nonoverlapping 100 bp windows, while the gene body range from TSS to TES was equally divided into 100 fractions. The average DNA methylation levels within every window or faction were calculated, and the mean value of each genomic locations type was then computed to profile the global methylation pattern around the genic region for every sample. In addition, the mean value of all samples in the sample group was used to profile the global methylation pattern around the genic region for each group. The methylation pattern was visualized by the R function “plot.”

### 4.13 DNA methylation levels within various genomic elements

The coordinate files of the known genomic elements were acquired from the UCSC Genome Browser, including Low_complexity elements, CpG island (CGI), gene body, 5′‐UTR, 3′‐UTR, three types of promoters (high‐CpG‐density promoters [HCP], intermediate‐CpG‐density promoters [ICP], and low‐CpG‐density promoters [LCP]) as previously defined,^72^, ^73^ and repetitive elements classified into seven categories: long terminal repeat (LTR), long interspersed elements (LINE), short interspersed elements (SINE), Retroposon_SVA, transposon, satellite, and microsatellite. The coordinate files of the imprinting control region (ICRs) were obtained from the paper of Hamada et al.^74^ Metastable epialleles (MEs) were obtained from the paper of Noah J. Kessler. et al.^75^ To evaluate the average DNA methylation level of each type of genomic element, only regions covering more than three CpG sites were reserved, and the mean methylation level of all retained CpG sites within a specific region was defined as the DNA methylation level of the corresponding region.

### 4.14 DNA methylation pattern of selected DMRs in preimplantation embryos

To calculate the methylation level of each intergenerationally correlated DMR mentioned in Figures S6 and S7, each DMR was expanded with additional 150 bp in both the upstream and downstream directions. Then, each single‐cell DNA methylation data recording methylation level of every CpG sites was used as input, and expanded DMRs covering more than three CpGs in DNA methylome data of early embryo development was reserved. The DNA methylation level of corresponding DMRs was calculated by averaging the methylation level of all retained CpG sites within a specific expanded DMR. Next, samples with at least one DMRs of not NA value were reserved and merged into single file. The average methylation value of every sample in different stages was calculated respectively, and defined as the methylation level of the target expanded DMR in each stage. Ward hierarchical clustering for each intergenerationally correlated DMR was performed using the function “hclust” in R package stats (version 3.6.0) based on the scaled average DNA methylation level in different stages of early embryo development.

### 4.15 RNA‐seq data analysis

The quality of the raw fastq data was first assessed by the FastQC tool (version 0.11.9), and then processed by the software TrimGalore with the parameter of “–quality 20 –stringency 3 –length 36 –paired” to remove the adapter, ploy‐N, inferior‐quality bases and reads less than 36 bases. Clean reads were then aligned to the *H. sapiens* reference genome (human GRCh38/hg38) using STAR software (version 2.7.8a) with default parameters. The uniquely aligned reads were subsequently counted by featureCounts software (version 1.6.3).^76^ Finally, DEseq2 (version 1.24.0) was applied to generate the normalized count matrix for offspring or mother samples. Detailed information on all RNA‐seq libraries is listed in Table S2.

### 4.16 Identification of differentially expressed genes

The intergroup differential expression analysis for either offspring or mother was performed using the R package DESeq2 (1.24.1).^77^ Genes meeting the criteria of fold change values more than 1.5 or less than 0.67 and *p* values less than .05 were defined as DEGs.

### 4.17 Principal component analysis and hierarchical clustering analysis

The average methylation value of the 200 bp bins around the entire genome was used for the hierarchical clustering analysis and PCA to evaluate the global DNA methylation similarity of offspring and parental samples, with the function “clusterSamples” in R package methylKit (version 1.10.0)^71^ and the function “pca” in R package pcaMethods (version 1.76.0),^78^ respectively. PCA was also performed to evaluate the transcriptome profile of offspring and mother samples with the function “pca” in the R package pcaMethods (version 1.76.0), using the normalized counts matrix for gene expression generated by DESeq2 (version 1.24.0). The distance matrices among RNA libraries were calculated using function dist with default parameters in the R package stats (version 3.6.0) and were visualized using the R package pheatmap (version 1.0.12). The classification of the dynamic pattern of DNA methylation during the development of preimplantation embryos and the dynamic pattern of gene expression during folliculogenesis and preimplantation embryos was based on the hierarchical clustering analysis, employing the function “dist” in the R package stats (version 3.6.0) and the function “hcluster” in the R package amap (version 08–18) with the parameter “method = pearson.”

### 4.18 Correlation analysis

The mean value of DNA methylation level of each remaining 200 bp bins was calculated for each group (AMA‐offspring, AMA‐mother, AMA‐father, Young‐offspring, Young‐mother, Young‐father), and the correlation coefficients and confidence intervals between AMA and Young groups were calculated by the function “stat_cor” in the R package ggpubr (version 0.4.0) with default parameters.^79^ The Spearman's correlation coefficients and confidence intervals between parents and offspring samples were calculated using the function “cor.test” and “cor” in R package stats (version 3.6.0) for either the methylation level of selected DMRs or the expression level of selected DEGs. Only DMRs or DEGs with a *p* value threshold of .05 and correlation Spearman's correlation coefficients (*R* value) greater than 0.6 were defined as candidate intergenerational related DMRs or DEGs.

### 4.19 Genomic functional annotation

The nearest genes, genomic features and the distance to the transcriptional start site (TSS) of DMRs were annotated using the R package ChIPseeker (version 1.20.0) and *H. sapiens* annotation package org.Hs.eg.db (version 3.8.2).^80^ The promoter was identified as the region from 3 kb downstream to 3 kb upstream of the TSS. The coordinate files of genomic elements were downloaded from the UCSC Genome Browser as mentioned in “***DNA methylation levels in specific genomic elements***.” The function “foverlaps” in R package data.table (version 1.14.0) was applied to identified the DMRs with at least 1‐bp overlap with any genomic elements.

### 4.20 Gene Ontology enrichment analysis

GO enrichment analysis for biological processes was performed to assess the potential biological functions of selected DMRs and DEGs, using the “enrichGO” function in the R/Bioconductor package “clusterProfiler (3.8.1)” packages

### 4.21 Statistical analysis

An unpaired two‐tailed *t*‐test in GraphPad Prism (Version 9.2.0) was applied to determine the significance of differences in the intergroup comparison of other clinical features for either parents or progenies. Fisher's exact test in SPSS (version 26.0) was used to determine the significance of differences in the comparative analysis of sexes and modes of birth. The statistical significance of Gene Ontology (GO) term enrichment analysis was determined using a hypergeometric test in the R package clusterProfiler, and the *p* value was adjusted by the multiple test adjustment (Benjamini–Hochberg, BH).

For bar‐dot plots of gene expression determined by qRT‐PCR, the significance of differences between two groups was determined by unpaired two‐tailed *t*‐test in GraphPad Prism (Version 9.2.0). For box‐dot plots of DNA methylation, the significance of differences between two groups was determined by the Wilcoxon rank‐sum test. The *p* value for the comparison among multiple groups was determined by the Kruskal–Wallis test. For a column graph of the proportion of bins with significantly differential DNA methylation levels for paternal or maternal samples, the chi‐squared test was applied to calculate the significance of differences between all 200 bp bin groups and correlated AMA‐DMRs of offspring groups. Unless otherwise stated, Spearman‐based correlation tests were used to determine the correlation coefficient and *p* value between any two groups (ns: *p* > = .05; ∗*p* < .05; ∗∗*p* < .01; ∗∗∗*p* < .001).

## CONFLICT OF INTEREST

The authors declare no conflict of interest.

## Supporting information

Supporting Information

Supporting Information

Supporting Information

Supporting Information

Supporting Information

Supporting Information

Supporting Information

Supporting Information

Supporting Information

Supporting Information

Supporting Information

Supporting Information

Supporting Information

Supporting Information

Supporting Information

Supporting Information

Supporting Information

Supporting Information

Supporting Information
