# Supplementary material for: The combination of DNA methylome and transcriptome revealed the intergenerational inheritance on the influence of advanced maternal age
Source: Clin Transl Med. 2022 Sep 14;12(9):e990. doi: 10.1002/ctm2.990 (PMC9473489; doi:10.1002/ctm2.990)
Supplement: Supplementary file 1 — Supporting Information [file CTM2-12-e990-s002.docx]

**Additional file 1. Containing Supplementary Table S1-Supplementary Table S9:**

**Supplementary Table S1**. Statistics for clinical features of families in RRBS and RNA-seq.

**Supplementary Table S2**. Summary for the sequencing information of all RNA-seq libraries and the RRBS, as well as the number and the average coverage depth of CpGs detected in different minimum coverage value, the overlap ratio (%) and the specific number of overlapped genomic elements for each RRBS library.

**Supplementary Table S3**. The list of AMA related DEGs for neonatal RNA-seq samples or maternal RNA-seq samples. DEGs: gene with pvalue < 005 and Fold change > =1.5.

**Supplementary Table S4**. The list of AMA related DMRs for paternal, maternal and neonatal RRBS samples. DMRs: 200bp bins with |differential DNA methylation value| >15% and q-value <0.05.

**Supplementary Table S5**. The representative enrichment gene ontology (biological process) terms for AMA related DMRs for paternal, maternal and neonatal groups.

**Supplementary Table S6**. The representative enrichment gene ontology (biological process) terms for DEGs of maternal or neonatal groups.

**Supplementary Table S7**. The representative enrichment gene ontology (biological process) terms for common AMA-related DEGs between maternal and neonatal groups.

**Supplementary Table S8**. The genomic locations and nearest genes for 91 intergenerationally correlated DMRs mentioned in figure **4D**.

**Supplementary Table S9**. The lists of different classes of overlapped AMA-related Upregulated-DEGs and downregulated-DEGs mentioned in figure **5A**.
