# Supplementary figures and images for: The combination of DNA methylome and transcriptome revealed the intergenerational inheritance on the influence of advanced maternal age
Source: Clin Transl Med. 2022 Sep 14;12(9):e990. doi: 10.1002/ctm2.990 (PMC9473489; doi:10.1002/ctm2.990)

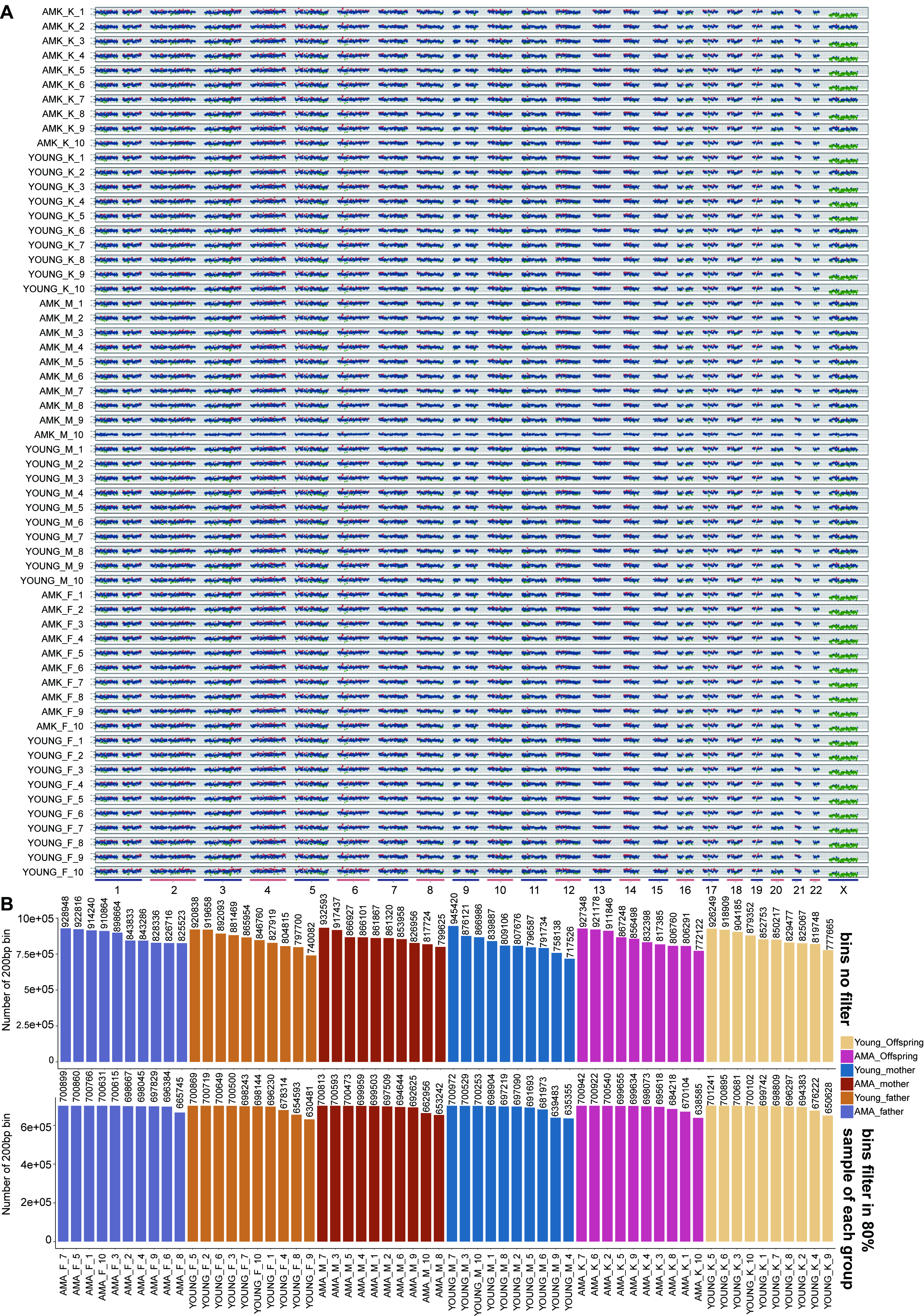

Supplement: Supplementary file 2 — Supporting Information [file CTM2-12-e990-s012.tif]

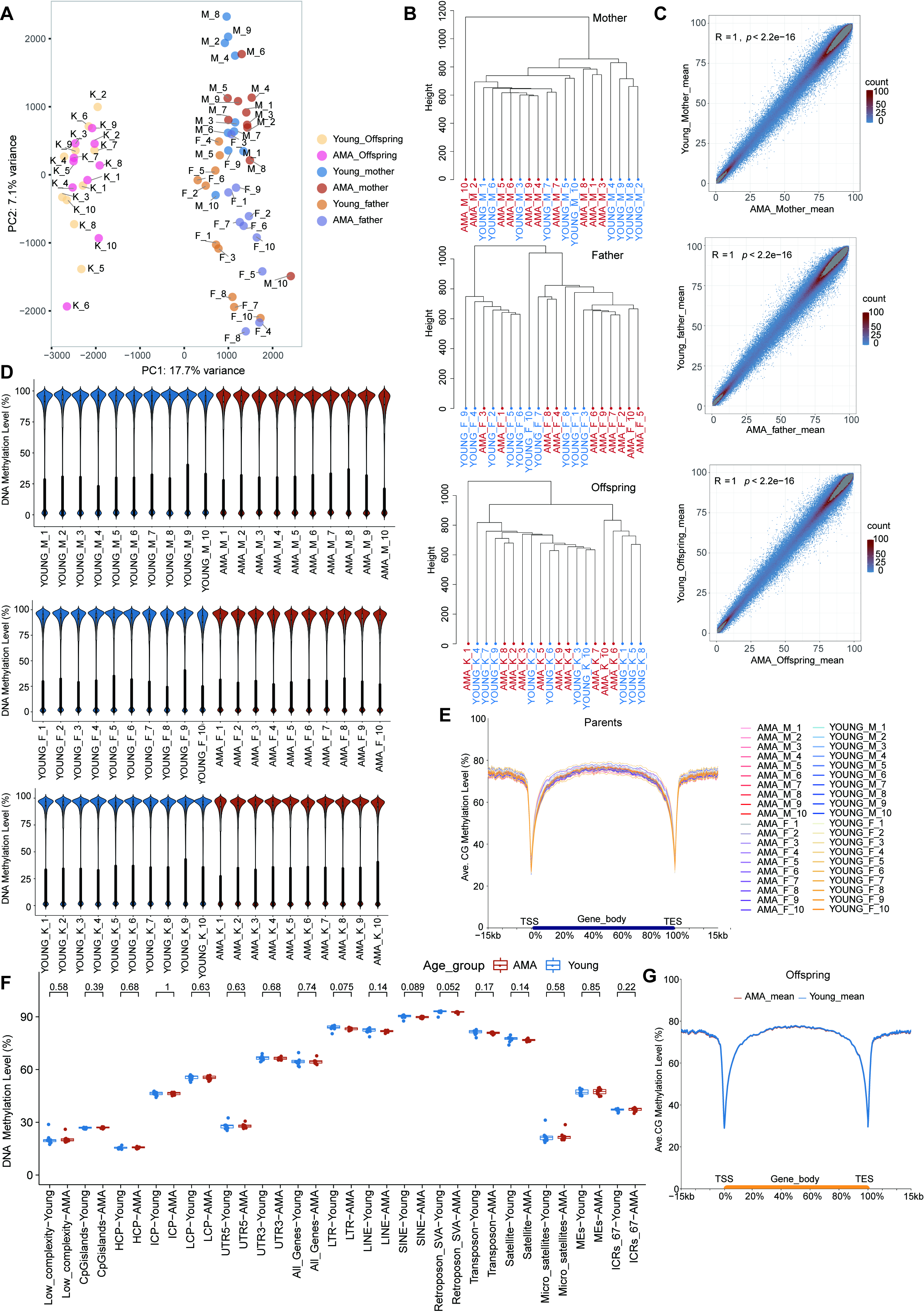

Supplement: Supplementary file 3 — Supporting Information [file CTM2-12-e990-s003.tif]

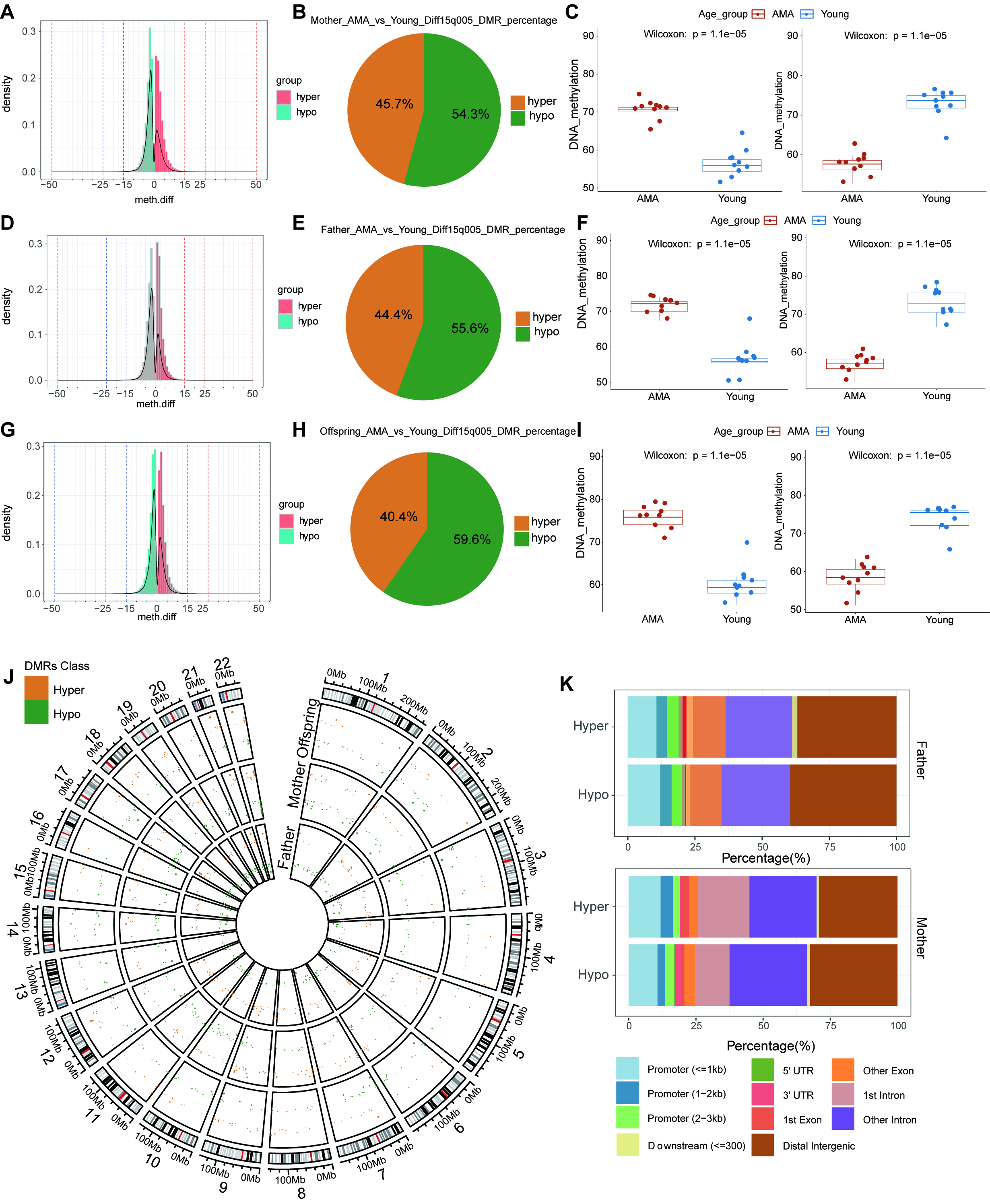

Supplement: Supplementary file 4 — Supporting Information [file CTM2-12-e990-s007.tif]

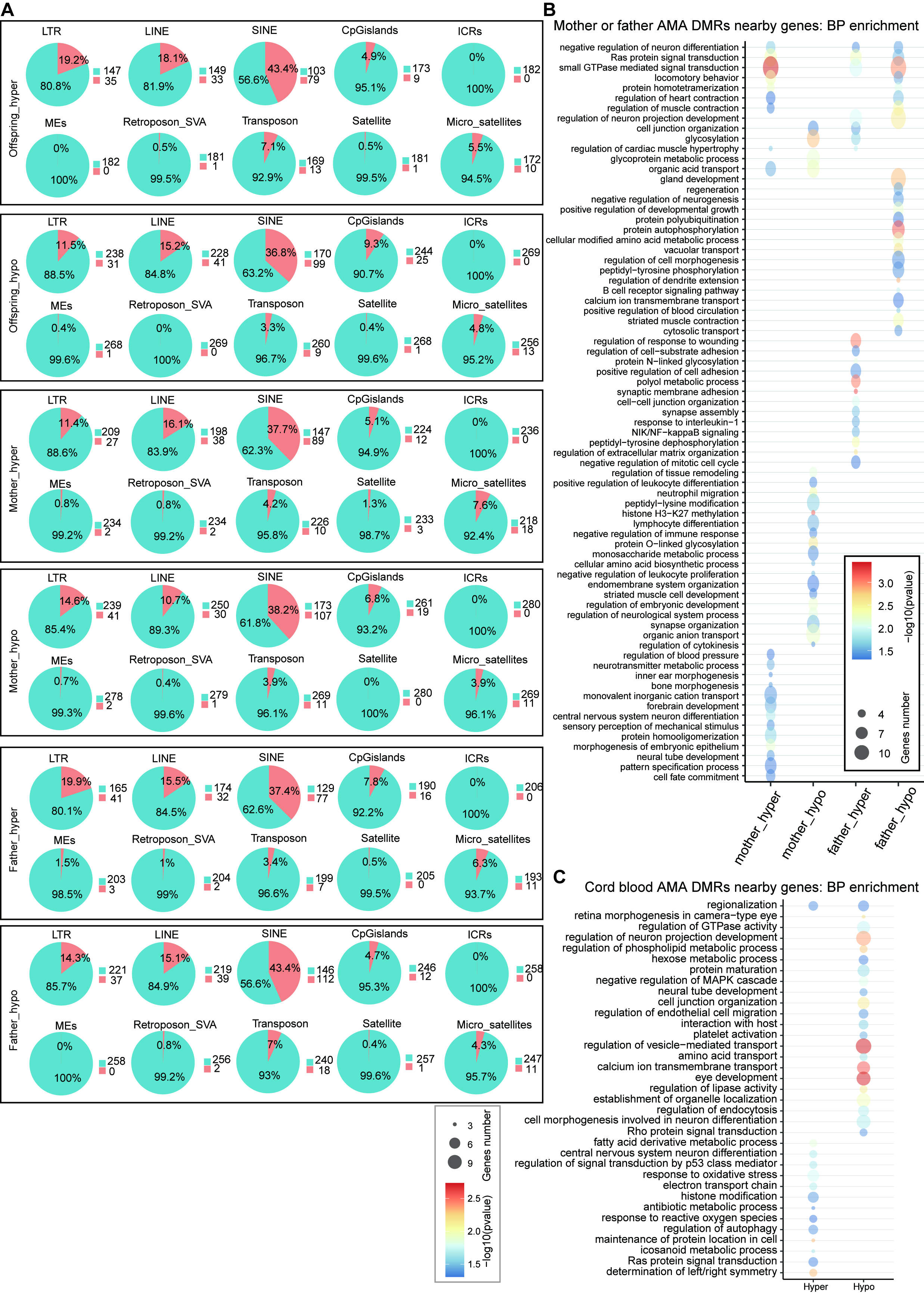

Supplement: Supplementary file 5 — Supporting Information [file CTM2-12-e990-s001.tif]

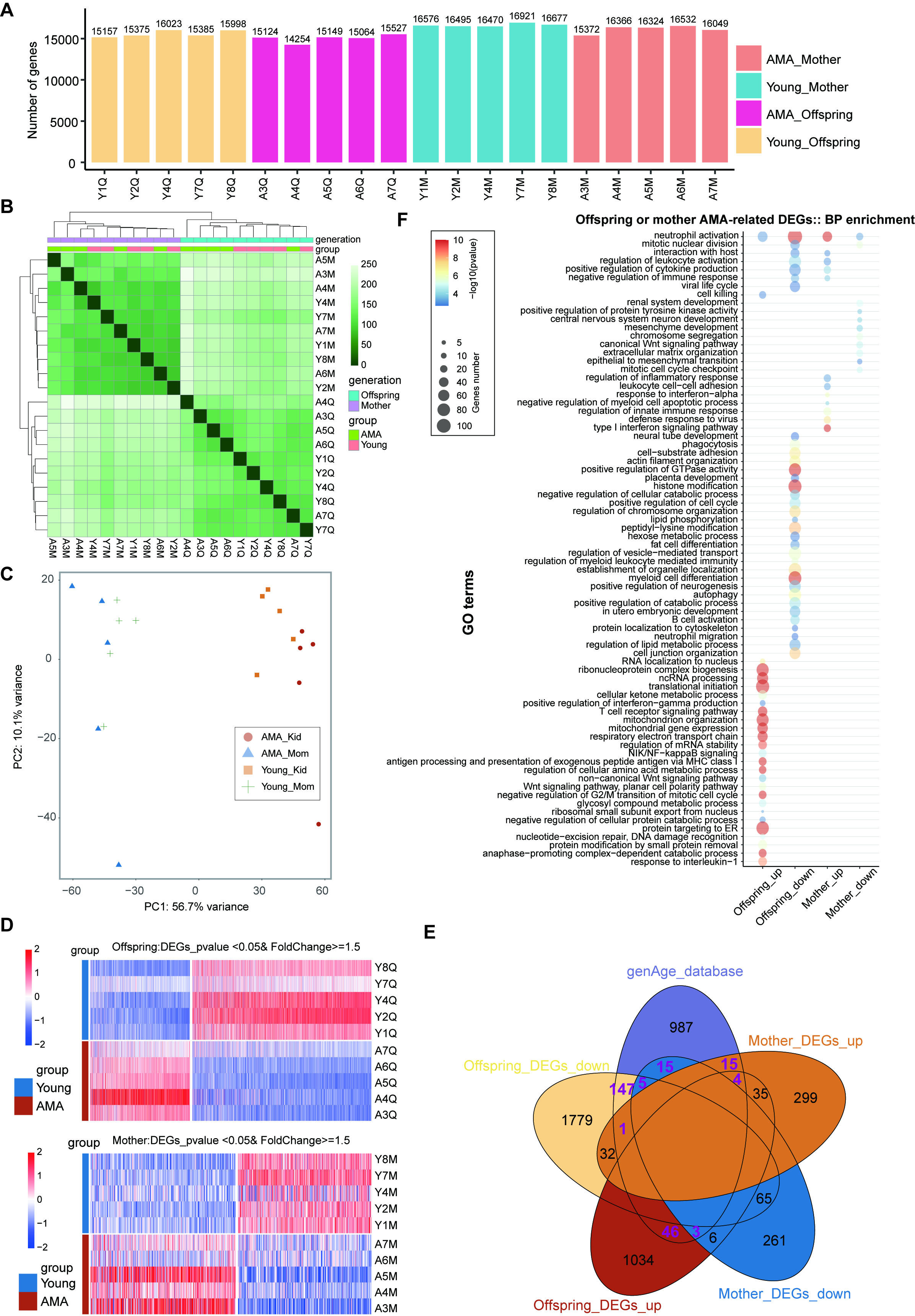

Supplement: Supplementary file 6 — Supporting Information [file CTM2-12-e990-s011.tif]

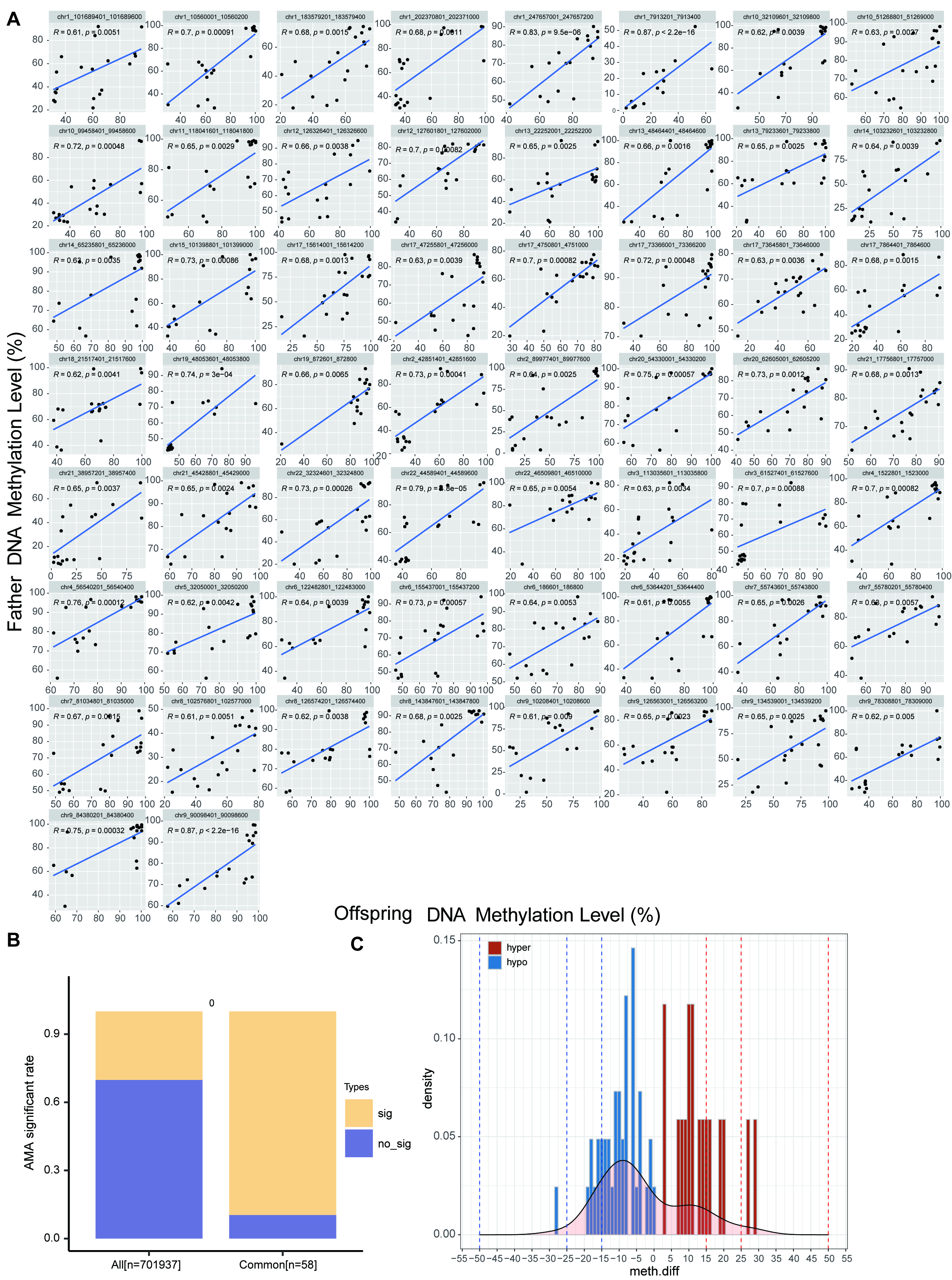

Supplement: Supplementary file 7 — Supporting Information [file CTM2-12-e990-s018.tif]

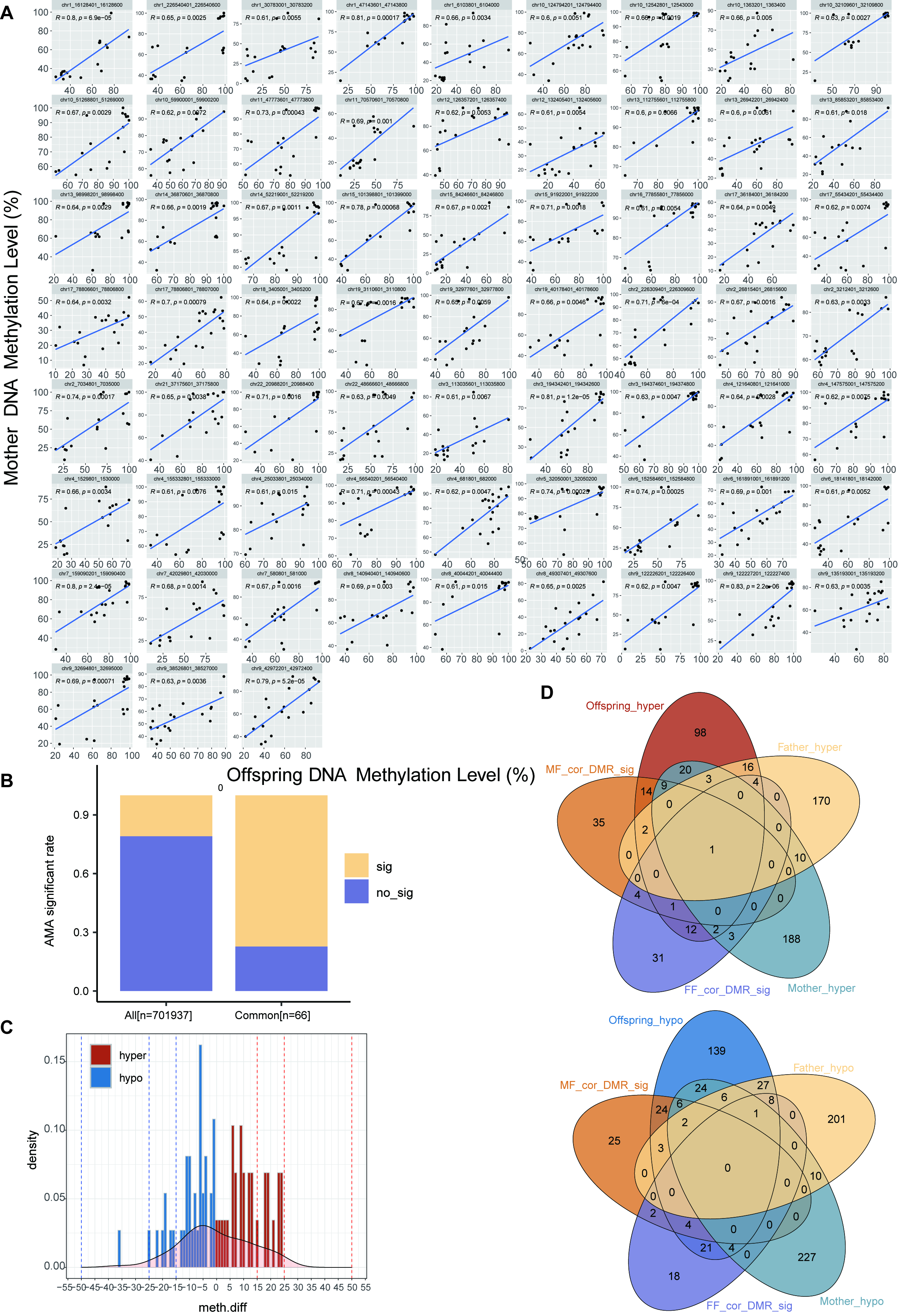

Supplement: Supplementary file 8 — Supporting Information [file CTM2-12-e990-s017.tif]

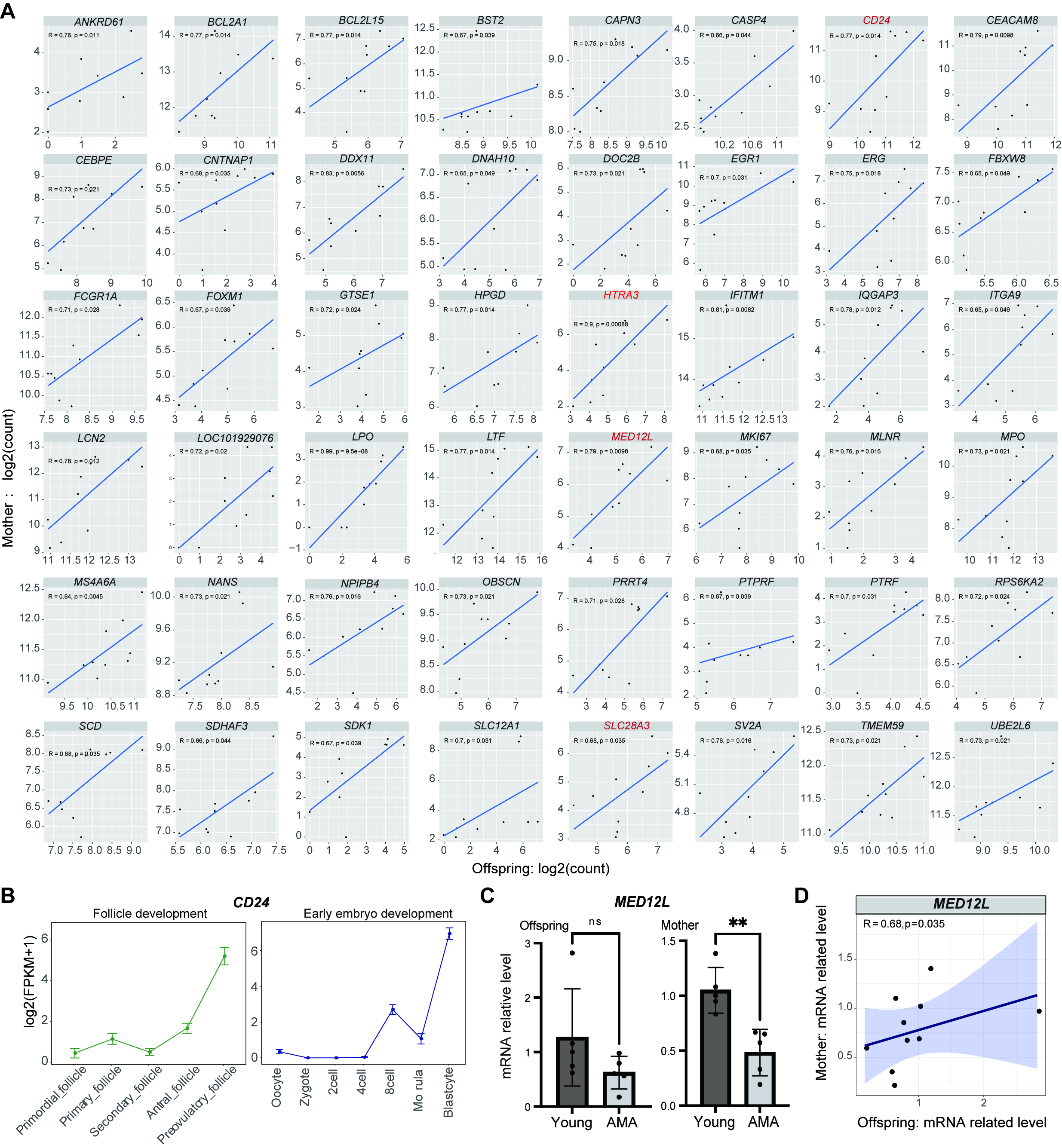

Supplement: Supplementary file 9 — Supporting Information [file CTM2-12-e990-s004.tif]
