# Supplementary material for: The combination of DNA methylome and transcriptome revealed the intergenerational inheritance on the influence of advanced maternal age
Source: Clin Transl Med. 2022 Sep 14;12(9):e990. doi: 10.1002/ctm2.990 (PMC9473489; doi:10.1002/ctm2.990)
Supplement: Supplementary file 19 — Supporting Information [file CTM2-12-e990-s015.docx]

**The combination of DNA methylome and transcriptome revealed the intergenerational inheritance on the influence of advanced maternal age**

Lingyue Hua ^a,b,c,d^, Wei Chen ^a,b,c,d,#^, Yan meng ^e,#^, Meng Qin ^a,b,c,d^ , Zhiqiang Yan ^a,b,c,d,^, Rui Yang ^a,b,c,d^, Qiang Liu ^a,b,c,d^, Yuan Wei ^b,f,g^, Yangyu Zhao ^b,f,g^, Liying Yan ^a,b,c,d,^* , Jie Qiao ^a,b,c,d,h,i,j,^*

^a^ Center for Reproductive Medicine, Department of Obstetrics and Gynecology, Peking University Third Hospital, Beijing 100191, China

^b^ National Clinical Research Center for Obstetrics and Gynecology (Peking University Third Hospital), Beijing 100191, China

^c^ Key Laboratory of Assisted Reproduction (Peking University), Ministry of Education, Beijing 100191, China

^d^ Beijing Key Laboratory of Reproductive Endocrinology and Assisted Reproductive Technology, Beijing 100191, China

^e^ Department of Obstetrics and Gynecology , Beijing Jishuitan Hospital ,Bejing 100096, China

*^f^ Department of Obstetrics and Gynecology, Peking University Third Hospital, Beijing, 100191, China*

*^g^ National Center for Healthcare Quality Management in Obstetrics, Beijing, 100191, China*

^h^ Beijing Advanced Innovation Center for Genomics, Beijing 100871, China

^i^ Peking-Tsinghua Center for Life Sciences, Peking University, Beijing 100871, China

^j^ Research Units of Comprehensive Diagnosis and Treatment of Oocyte Maturation Arrest, Beijing 100191, China

*Corresponding authors.

*E-mail addresses*: [jie.qiao@263.net](mailto:jie.qiao@263.net) (J. Qiao); [yanliyingkind@aliyun.com](mailto:yanliyingkind@aliyun.com) (L. Yan).

^#^ These authors contributed equally to this work.

**ABSTRACT**

The number of women delivering at advanced maternal age (AMA; >=35) continuously increasing in developed and high‐income countries. Large cohort studies have associated AMA with a significantly increased risk of various pregnancy complications and adverse pregnancy outcomes, which also raises great concerns about the adverse effect of AMA on the long-term health of the offspring. Specific acquired characteristics of parents can be passed on to descendants through certain molecular mechanisms, yet the underlying connection between AMA-related alterations in parents and offspring remains largely uncharted. Here, by profiling the DNA methylomes and transcriptomes of paired parental peripheral blood and cord blood samples from nuclear families, we revealed that AMA would induce a series of aging-like changes in the DNA methylome and gene expression in both parents and offspring. The expression changes in several genes, such as *SLC28A3*, were highly relevant to the disorder in DNA methylation. In addition, AMA-related differentially methylated regions (DMRs) identified in the mother and offspring groups showed remarkable similarities in both genomic locations and biological functions, mainly involving neuron differentiation, metabolism, and histone modification pathways. The AMA-related differentially expressed genes (DEGs) shared by mother and offspring groups were highly enriched in the processes of immune cell activation and mitotic nuclear division. We further uncovered the developmental-dependent dynamics for the DNA methylation pattern of those intergenerationally correlated DMRs during pre-implantation embryonic development, as well as the diverse gene expression patterns during gametogenesis and early embryonic development for those common AMA-related DEGs presenting intergenerational correlation, such as *CD24*. Moreover, some intergenerational DEGs, typified by *HTRA3*, also showed the same significant alterations in AMA MII oocyte or blastocyst. Our results revealed the potential intergenerational inheritance of both DNA methylome and transcriptome changes associated with AMA and provided new insights relevant to understanding health problems in AMA offspring.

**Keywords:** Advanced maternal age, Intergenerational inheritance, DNA methylation, Transcriptome

**1 I****NTRODUCTION**

Advanced maternal age (AMA) is defined as a maternal age of 35 years or older at the time of delivery[1]. During the past three decades, the percentage of AMA mothers has rapidly increased in many developed and high‐income countries, reaching 23% in the United States in 2014 and as high as 33.4% in Korea in 2019 [1, 2]. However, aging, as an inevitable biological progress, comes with the accumulation of organ functional decline and cell damage, such as cardiovascular homeostasis disruption, systemic inflammation, mitochondrial dysfunction and so on [3, 4]. Human plasma proteome research further revealed nonlinear changes during the aging process, with one noticeable crest of protein expression changes around age 34 [5]. In fact, AMA is well recognized as a major risk factor for various pregnancy complications and adverse pregnancy outcomes including preeclampsia, gestational diabetes mellitus, miscarriage and preterm delivery [6, 7].

Numerous studies have indicated that specific parental environmental experiences and physiological changes, such as stress and starvation, could be engraved in nongenetic mechanisms and transmitted to progeny, thereby influencing their phenotypes [8]. Aging-related alterations are also reflected by various molecular hallmarks, including genomic instability and increased aberrant transcriptional and DNA methylation . The DNA methylome pattern of a specific set of CpGs is even referred to as the “epigenetic clock” for its accuracy in estimating biological age [9]. Previous studies have revealed that global epigenetic reprogramming during gametogenesis and embryo development remodel epigenetic patterns [10, 11]. However, 20~30% of DNA methylation can escape from the first epigenetic reprograming during preimplantation embryo development, which is regarded as one important mechanism of epigenetic intergenerational inheritance to pass on the specific parental features to the offspring [12, 13]. Only a few of parental DNA methylation patterns are able to avoid the much stronger second round of epigenetic reprogramming that occurs during gametogenesis [11]. This may mediate the inheritance of some special phenotypes over multiple generations, a process defined as the epigenetic transgenerational inheritance [13]. Consistent with this, previous studies have pointed out that parental age might disturb different characteristics of offspring [14], such as the famous "Lansing effect", which describes the shorter lifespan of the offspring of older parents [15], and abnormal offspring behavioral phenotypes associated with DNA methylation abnormalities in sperm from older fathers [16]. In particular, women over the age of 35 generally suffer from a dramatic decrease in fecundity [17]. Apart from a well-documented increase in aneuploidy[18], AMA has been reported to be accompanied by dysregulation of both the DNA methylome and transcriptome inoocytes [19-25], as well as an overall decrease in gene expression in blastocysts [26]. Meanwhile, researchers have also claimed that offspring delivered by AMA mothers would face higher risks of autism and impaired cardiometabolic health [27, 28].

Unexpectedly, studies on the epigenomic and transcriptomic impacts of AMA on either pregnant women or offspring are very scarce [29, 30], and discussions about the concrete relationships of molecular alterations in parents and offspring are even less well studied. In fact, to date, only three research groups have identified CpG sites that were correlated with maternal age, in neonatal heel blood, cord blood and the peripheral blood of adult daughters, separately [31-33]. Hereby, we performed integrated analysis of the transcriptome and DNA methylome of nuclear families and revealed the potential phenomenon of intergenerational inheritance of AMA-related alterations in both the DNA methylome and transcriptome. We also confirmed that specific abnormal changes in offspring might originate from the disturbance of oocytes or embryos by AMA. Hence, our data provide valuable resources and new insights for understanding the molecular mechanisms underlying the influence of AMA and interpreting the health problems in the later life of AMA offspring.

**2 RESULTS**

**2.1 AMA-related abnormal changes in the parental DNA methylome**

To investigate the influence of AMA on the DNA methylome of parents and offspring, we applied reduced-representation bisulfite sequencing (RRBS) to umbilical cord blood (UCB) samples and corresponding parental peripheral blood (PPB) samples from nuclear families. Those samples were further classified into AMA and Young groups based on maternal age (AMA: maternal age of 36~43years old; Young: maternal age of 22~29 years old) (Figure **1A**). There were no significant differences in maternal BMI or any clinical features of offspring between the AMA and Young groups (see Supplementary Table **S1** for more details). The genome-wide copy number variations (CNVs) were normal in all samples (Supplemental Figure **S1A**). The genome was then split into 200 bp bins, and only bins containing more than three CpG sites and conserved in 80% of the samples in each group were used for downstream analysis (Supplemental Figure **S1B**).

Principal component analysis (PCA) and hierarchical clustering analysis showed no obvious separation of the AMA and Young groups in either maternal or paternal samples (Figure **1B** and Supplemental Figure **S2A-2B**). The global patterns of DNA methylation levels were also similar among samples (Supplemental Figure **S2C-2D**). The mean value was approximately 75% in each group, and it was slightly lower in the AMA group than in the Young group for both maternal and paternal samples, but the difference did not reach statistical significance (Figure **1C** and **1D**). Meanwhile, the overall DNA methylation level around the gene body was similar among the four groups (Figure **1D**). Interestingly, it was notable that the average methylation levels of all four kinds of retrotransposons (including LTR, LINE, SINE and SVA) were significantly lower in AMA-mother than in Young-mother, while no differences were observed in the father group (Figure **1E** and Supplemental Figure **S2F**).

We next performed intergroup comparisons to elucidate the definitive influence of AMA, and identified 516 and 464 AMA-related DMRs in the mother and father groups, respectively (|df|>= 15% and q value < 0.05; for mothers: hyper DMRs (45.7%), n=236; hypo DMRs (54.3%), n=280; for fathers: hyper DMRs (44.4%), n=206; hypo DMRs (55.6%), n=258) (Figure **1F** and Supplemental Figure **S3A-S3F**; see Supplementary Table **S4** for more details). These DMRs were broadly distributed on the genome scale and primarily located outside of the promoter regions (Supplemental Figure **S3J-S3K**). Further functional genomic annotation revealed that a large percentage of DMRs were located in SINEs (37.4%~43.4%), LTRs (11.4%~19.9%), LINEs (10.7%~16.1%), as well as CpG islands (4.7%~7.8%) (Supplemental Figure **S4A**). However, mother-DMRs and father-DMRs were quite distinct in terms of their exact genomic positions, with only 14 hyper-DMRs and 19 hypo-DMRs in common (Figure **1G**). Searching for the nearest genes of DMRs in both longevity and aging databases [34, 35] revealed that 44 genes in the mother group and 20 genes in the father group had been reported to be associated with aging (Figure **1H** and **1I**). The nearest genes of two mother-DMRs, *RPS6KA5* and *ETS2*, were previously defined as senescence-associated secretory phenotype (SASP) genes[36] (Figure **1J**), while no overlap existed between the nearest genes of father-DMRs and SASP genes (Figure **1J**). Gene Ontology (GO) enrichment analysis revealed that the nearest genes of mother-DMRs were enriched in processes involving organ morphogenesis, histone modification, phospholipid metabolism, and leukocyte differentiation. However, the nearest genes of father-DMRs were highly enriched in wound healing, cell-substrate adhesion, and calcium ion transmembrane transport (Supplemental Figure **S4B**). Both mother-DMRs and father-DMRs were enriched in the processes associated with neuron differentiation (nearest genes: *CCND1*, *SPOCK1*), protein homotetramerization (nearest gene: *USP16*) and glycosylation (nearest genes: *GAL3ST1*) (Figure **1G** and Supplemental Figure **S4B**; see Supplementary Table **S4** for details). Together, the above results indicated that aging-related alterations in the parental DNA methylomes were present in AMA pregnancy and were more conspicuous in the maternal part.

**2.2 AMA induced DNA methylation changes in offspring**

Then, we analyzed the DNA methylomes of UCB samples to evaluate the epigenetic changes in AMA offspring. Similar to what was observed in PPB, PCA and hierarchical clustering analyses showed no obvious separation of the AMA and Young groups (Figure **2A** and Supplemental Figure **S2A**). The mean value showed no significant intergroup difference (Young: 75.31%; AMA: 74.99%; *P* value = 0.44; Figure **2B**), and the average DNA methylation pattern on the genome-wide scale or specifically around gene bodies was comparable between the AMA and Young groups. (Figure **2C** and Supplemental Figure **S2C-2D**, **S2G**). Nevertheless, the average DNA methylation levels of CpG islands significantly decreased in the AMA group (Young: 26.52%; AMA: 26.50%; *P* value = 0.035; Figure **2D**). Intergroup comparison analysis further identified 182 hyper DMRs and 269 hypo DMRs (|df|>= 15% and q value<0.05) (Figure **2E**, Supplemental Figure **S3G-S3I**), which were broadly distributed on all chromosomes and mainly in nonpromoter regions (Figure **2F** and Supplemental Figure **S3J**). Once again, we observed a larger percentage of hypo DMRs than hyper DMRs (40.4% versus 59.6%, Supplemental Figure **S3H**) and a high proportion of hyper/hypo DMRs located in SINE (36.8% and 43.4%), LTR (11.5% and 19.2%), LINE (15.2% and 18.1%) (Supplemental Figure **S4A**), as described for the parental sample.

Among the nearest genes of offspring-DMRs, 25 genes were listed in the longevity or aging databases (Figure **2G**). Remarkably, the SASP gene *RPS6KA5* was also observed among them, with a nearby hypo DMRs located in the intron 1 region (chr14: 91033401-91033600) (Figure **2H**). As shown in **Figure** **1J**, the same region was also identified as AMA-related hypo DMR in the mother group*.* GO analysis suggested that disturbance of those DMRs might influence various processes associated with vesicle-mediated transport, autophagy, electron transport chain, eye development, and so on (Supplemental Figure **S4C**). Importantly, quite a few GO terms were also found in the enrichment analysis of the mother group, including processes involving neuron differentiation, regulation of GTPase signal transduction, metabolism, and histone modification (Supplemental Figure **S4B and S4C**). Integrated analysis between parental DMRs and offspring-DMRs further uncovered overlapping common DMRs that shared the same trend between offspring and parents (MK_common DMRs: n=64; FK_common DMRs: n=58, FMK_common DMRs: n=13) (Figure **2I**). Taken together, our findings indicated that AMA would also disturb the DNA methylome of the offspring. These changes bore many remarkable similarities to those in the maternal DNA methylome.

**2.3 AMA altered the** **maternal and** **neonatal** **transcriptomes**

In addition to examining the DNA methylome, we also performed mRNA-seq of maternal peripheral blood and UCB samples to explore the influence of AMA on gene expression (Supplementary Table **S1** and Supplemental Figure **S5A**). Similar to the observations in the DNA methylome, correlational analysis and PCA based on the transcriptomes of all samples showed obvious separation of the mother and offspring groups but only slight separation between the AMA and Young groups (Supplementary Figure **S5B** and **S5C**). Intergroup comparison analysis identified 741 and 3157 DEGs in the mothers and offspring, respectively (Figure **3A** and **3B,** Supplementary Figure **S5D** and **S5E**; p < 0.05 and fold change >=1.5; for mothers: Up-regulated DEGs: n=386 & Down-regulated DEGs: n=355; for offspring: Up-regulated DEGs: n=1128 & Down-regulated DEGs: n=2029; see Supplemental Table **S5** for details). A total of 206 DEGs in the offspring group and 43 DEGs in the mother group were recorded in the longevity or aging databases (Supplementary Figure **S5E**). GO analysis showed that bothoffspring-DEGs and mother-DEGs were involved in neutrophil activation, mitotic nuclear division, and neuron development. Offspring-DEGs also focused on those processes related to embryo and placenta development, protein modification, regulation of RNA processing and translation, and mitochondrial function, while mother-DEGs were highly enriched in mesenchyme development, regulation of inflammatory response, chromosome segregation, and cell−cell adhesion (Supplementary Figure **S5F,** see Supplemental Table **S6** for details).

A total of 109 common AMA-DEGs shared the same trend between the mother and offspring groups (Figure **3C**, common Up-regulated DEGs: n=39 and common down-regulated DEGs: n=70).  *GMFG,* as a SASP gene within this set, was upregulated in the AMA group (Figure **3C**). GO enrichment analysis suggested that these DEGs might be involvedin the processes of cytokine production, cell mitosis, protein modification, neutrophil activation, defense response to gram−negative bacterium and the NF-KB signaling pathway (Figure **3D;** see Supplemental Table **S7** for details). Overall, the above results revealed that the fluctuation in the transcriptome reflected the aging-like effect of AMA on both offspring and mothers, which shared many similarities with DMRs in related biological processes. The results also presented considerable similarities in the transcriptional alterations between the mother and offspring.

We then performed an integrated analysis between DEGs and DMRs for either mother or offspring to identify DEGs potentially rooted in the DNA methylation change of nearby DMRs (offspring-DEGs, n= 58; mother-DEGs, n=13) (Figure **3E**, **3F** and **3G**). Specifically, *SLC28A3*, a gene encoding a nucleoside transporter, was significantly downregulated in both the offspring and mothers of the AMA group, along with an increased DNA methylation level in DMRs located in intron 1(Figure **3E**, **3F**, **3G** and **3H**). The reduced expression of *SLC28A3* was further validated by real-time fluorescence quantitative polymerase chain reaction (qRT-PCR) (Figure **3I**). This result suggested the highly interconnected changes in the transcriptome and DNA methylome in the AMA group.

**2****.4** **Specific alterations in AMA offspring presented intergenerational correlation**

Although common changes between parents and offspring have been observed in both the transcriptome and DNA methylome, how close the connection between the changes in the offspring and parents is remains uncertain. In fact, the *CD24* (CD24 molecule), one of the common DEGs and encoding a glycosylphosphatidylinositol-linked cell surface protein tightly correlated with cell pluripotency [37], was not only significantly downregulated in both AMA groups, but also showed strong linear corrections between paired mother and offspring (Supplementary Figure **S8A**), which were validated using qRT-PCR, too (Figure **4A** and **4B**). This observation hinted that part of alterations in offspring might be directly correlated with the parental changes induced by AMA. Thus, benefiting from the definite parentage in our cohort, we calculated the Spearman correlation coefficients between parents and offspring for both offspring-DMRs and offspring-DEGs to identify the intergenerationally correlated DMRs (R >= 0.600 and value < 0.05; Supplementary Figure **S6** and **S7**) and DEGs (R >= 0.600 and p value < 0.05; Supplementary Figure **S8A**). Among these, 18 and 14 DMRs respectively belonged to common mother-offspring and father-offspring DMRs shown in Figure **2I** (23% and 19%)**,** while 48 DEGs were previously identified as common DEGs showed in Figure **3C** (44%; Up-DEGs: n=11; Down-DEGs: n=37; Figure **4C**). This result suggested that a considerable proportion of AMA-related DMRs and DEGs observed in offspring might be directly inherited from parents.

The parental characters were passed on to offspring mainly through the gamete and embryo, and previous studies have outlined highly diverse molecular dynamics for different genomic elements and various genes during gametogenesis and pre-implantation development [12, 38-40]. To investigate the DNA methylation pattern of those intergenerationally correlated DMRs during the epigenetic reprogramming process, we here reanalyzed the published single-cell chromatin overall omic-scale landscape sequencing (scCOOL-seq) data of human pre-implantation embryonic development [41] and depicted the dynamic DNA methylome patterns for 91 intergenerationally correlated DMRs detected in the data (Figure **4D** and Supplementary table **S8**). Those DMRs were further classified into seven clusters using hierarchical clustering, and the dynamic pattern in each cluster showed strong development-stage specificity (MeE1-MeE7, Figure **4D**). It indicated that DNA methylation patterns of those DMRs were strictly regulated by demethylation and remethylation mechanism during embryonic development, rather than just keep out of the epigenetic reprogramming process.

Meanwhile, we also downloaded single-cell transcriptional data of human oogenesis and pre-implantation embryos to profile the expression dynamics of 48 intergenerationally correlated common DEGs [38, 40]. Among the 10 patterns defined in follicle generation, genes with the RO2 pattern drastically increased from secondary follicle to antral follicle, a phenomenon that might play important role in follicular lumen formation. Genes with the RO4 and RO5 patterns showed the opposite pattern and striking changes in the preovulatory stage. In particular, Suzhen Yuan and his colleagues found that the upregulation of EGR1 (RO4 pattern) participated in granulosa cell apoptosis and follicle atresia during ovarian aging [42]. In addition, RO3, RO6, and RO9 patterns presented drastic fluctuations in gene expression from the primordial follicle stage to the primary follicle stage (Figure **4E**). Six distinct clusters of gene expression patterns were identified in preimplantation embryos (Figure **4F**). The dramatically increase in expression after the morula stage implied that genes with the RE1 pattern might be associated with the cell differentiation of blastocyst. The specific high expression after the 4-cell stage evinced the character of zygotic genes after major ZGA for genes with the RE2 and RE6 patterns. In contrast that of RE2 gene, the high expression levels of genes with the RE3 pattern decreased sharply after the 4-cell stage, implying that those genes might be potential maternal-effect genes that were largely degraded during the maternal-to-zygotic transition (MZT). It is worth noting that the expression of *CD24* both increased sharply during the transition from the antral follicle to the preovulatory follicle during folliculogenesis, echoing the early report about its important role in the regulation of ovulation [43]. During the development of early embryos, the expression of *CD24* was increased in the periods of ZGA and the transition from morula to blastocyst, (Figure **4F**), consistent with its high expression in the following villus trophoblast cell and the key roles in mediating immune tolerance at the fetal-maternal interface [44]. Together, these results revealed that the DNA methylation patterns of intergenerationally correlated DMRs and the expression patterns of those intergenerationally correlated DEGs varied during oocyte maturation and preimplantation embryonic development, and suggested the potential impact of AMA on multiple key processes in these two periods.

**2.5** **AMA-related transcriptional alterations existed in AMA oocyte and blastocyst.**

Several previous studies have discussed the influence of AMA on human MII oocytes or embryos after cryopreservation [45-47]. We then compared offspring-DEGs and mother-DEGs with two published blastocyst-DEG lists [26, 46] and one MII oocyte-DEG list [47], and revealed a series of overlapping DEGs (Figure **5A**). Among common AMA-DEGs between mother and offspring groups, two upregulated genes and two downregulated genes were observed in the oocyte, while 18 genes were downregulated in the blastocyst. Meanwhile, 11 of those 22 genes (*HTRA3*, *FCGR1A*, *BST2*, *MED12L*, *SLC12A1*, *MLNR*, *CNTNAP1*, *NPIPB4*, *CAPN3*, *DNAH10*, and *LOC101929076*) were previously identified as mother-offspring intergenerationally correlated DEGs (Figure **5A** and Supplemental Figure **S8A**). In particular, the *HTRA3* (high temperature requirement factor A3), which encodes a serine protease and has been reported to negatively regulate trophoblast invasion [48], has a reduced expression in the AMA mother, offspring and blastocyst groups (Figure **5A**, **5B** and **5C**)*.* In folliculogenesis, the expression of *HTRA3* increased sharply during the transition from the antral follicle to the preovulatory follicle, consistent with the previous report about the important role in the regulation of ovulation and luteinization [49]. In the development of early embryos, the expression of *HTRA3* was downregulated soon after fertilization (Figure **5D**). qRT-PCR further validated the significant downregulation in the AMA group and linear corrections between mother and offspring for *HTRA3* (Figure **5E** and **5F**)*.* Among the four DEGs observed in oocyte, *MED12L,* one mother-offspring intergenerationally correlated DEGs downregulated in the AMA group, showed a trend toward downregulation in both mothers and offspring in qRT-PCR validation, but statistical significance was reached only in the mother group (Supplementary Figure **S8B** and **S8C**). In conclusion, these results supported the view that some of the intergenerationally inherited alterations observed in the transcriptome of offspring directly came from the disturbance of AMA in oocytes or preimplantation embryos (Figure **5G** and **5H**).

**3. D****ISCUSSION**

A variety of socioeconomic factors in contemporary society have combined to produce an increase in pregnancies among women of advanced maternal age [50]. In addition to being an independent risk factor for various pregnancy complications, AMA is generally accompanied by greater risks of adverse pregnancy outcomes and adverse effects on the long-term health of offspring [27]. Although accumulating evidence has blamed offspring’s health issues on preexistent AMA-induced maternal abnormities, the underlying molecular mechanisms for the intergenerational hereditary phenomenon are still largely uncharted. In this study, we systematically profiled the influence of AMA on the DNA methylome and transcriptome of nuclear families and explored the potential origin of offspring changes, which might be inherited through gametes and embryos. We observed a significant reduction in the DNA methylation levels of various retrotransposons in the AMA-mother group, and on the CpG island in the AMA-offspring group. Many genes near AMA-DMRs, as well as AMA-DEGs, have been reported to be associated with aging. We also identified a series of genes whose expression changes might result from a corresponding alteration in DNA methylation and many common biological processes enriched for both AMA-DMRs and AMA-DEGs. This reflected the aging-like alteration induced by AMA and the consistency of AMA impacts across different omics. Furthermore, we highlighted the similar influence of AMA between parents and offspring, especially between mother and offspring. Combined with published datasets, our analyses further revealed the diverse methylation patterns of intergenerationally correlated DMRs and expression patterns of intergenerationally correlated DEGs presented in human folliculogenesis and preimplantation embryonic development. In particular, some AMA-DEGs were already significantly changed in MII oocyte or blastocyst, such as *HTRA3*, whose differential expressions was further verified in both the mother and offspring groups. In summary, this research unveiled the intergenerational relatedness of the alterations induced by AMA in both the DNA methylome and transcriptome and supported the claim that the adverse impact of AMA on the mother might interfere with oocytes and early embryos, and thus lead to abnormal changes in offspring.

Specific environmental exposures or experiences and physiological changes, such as starvation, depression and the aging process, could influence the stability of the DNA methylome[8]. HeiJmans and colleagues identified that the DNA methylation changes induced by starvation in the imprinting control region (ICR) near the *IGF2* gene in the mother were associated with the lowered birth weight in offspring [51]. The accumulation of errors in DNA methylation maintenance during the aging process generally leads to global decreases in DNA methylation [52]. Puberty and menopause demarcate the beginning and the end of the female reproductive life cycle, while 35 years of age is generally regarded as a turning point for female fecundity, marked by rapid declines of antimullerian hormone (AMH), ovarian reserve and increased risks of adverse pregnancy outcomes [53]. We observed significant alterations in a range of genes and genomic regions previously reported to be associated with the aging process, longevity and the senescence-associated secretory phenotype (such as *RPS6KA5* and *ETS2*) in AMA mothers. Many AMA-DEGs and AMA-DMRs were highly enriched in immunity processes and the processes of glycometabolism and cardiovascular function. Higher chronic proinflammatory status has been regarded as one prominent feature of aging [54]. Meanwhile, aging is well known as a major risk factor for metabolic syndrome, arteriosclerosis and cardiovascular disease[3]. The above observations together claimed a more universal aging process laying behind the reproductive aging in AMA mothers. Furthermore, this might partly explain the strongly independent association between AMA and the increased risk of gestational diabetes mellitus and preeclampsia [6]. The DNA methylome of AMA fathers also exhibited a series of alterations, which were enriched in the regulation of neuron projection development and response to wounding, and the number of AMA-related DMRs identified in fathers was similar to that identified in the mothers. Additional intergenerational contributions from AMA father might partly explain the significantly greater number of offspring-DEGs compared with mother-DEGs. A 10-year birth cohort study in Sweden have suggested a relationship between advanced paternal age and increased incidence of autism in the offspring [55]. Kaichi Yoshizaki and colleagues further showed that hypomethylation of mouse sperm DNA could alter the expression of genes in REST/NRSF pathway and induce an intergenerational influence on the neurodevelopmental programs in the offspring [56]. Thus, although the variations in AMA fathers showed less consistency with the aging process, advanced paternal age might also introduce specific intergenerational influence on offspring.

More importantly, we observed significantly downregulated methylation levels at retrotransposons (LTR, LINE, SINE, SVA) in the AMA-mother group. Retrotransposons make up more than one-third of the human genome, and show increased expression levels but decreased DNA methylation levels during aging in multiple species [57]. Our results further demonstrated that AMA might introduce an aging-like influence into the maternal DNA methylome. Nevertheless, the methylation levels of retrotransposons showed no significant inter-group difference infathers, although there was a larger age difference between fathers in the two groups. Recent genome-wide DNA methylation profiling studies further highlighted the sexual dimorphism in the specific age-related DNA methylation patterns of CpGs [58]. Females generally outlive males and have a younger epigenetic age than their male counterparts [59]. Previous studies discovered that sex hormone fluctuations can respond to changes in epigenetic age within a relatively short time in females [60, 61]. Thus, the much more dramatic changes in sex hormone levels before and after age 35 in females may be the immediate cause of the obvious sexual dimorphism in the methylation changes of retrotransposons. Due to the limited retrotransposon coverage of RRBS on coverage on retrotransposons, it would be better to use WGBS or pyrosequencing to verify the sexual dimorphism of the global DNA methylation change in retrotransposons in the future.

AMA might lead to premature senescence in the term placenta and first trimester villi of either human or mice [62]. Our study found that 25 genes near AMA-DMRs and 206 DEGs in AMA-offspring were associated with aging or longevity. Remarkably, the changes in DNA methylation or gene expression of several aging-related genes, such as *RPS6KA5* and *GMFG*, in AMA-offspring coincided with the alterations in AMA-mothers. Those molecular characteristics in AMA-offspring indicated that the aging-like influence of AMA was not limited to extraembryonic tissue. To date, three studies using methylation microarrays have suggested alterations in offspring DNA methylation related to maternal age in childbirth. Markunas et al. claimed that methylation changes in 5 identified CpGs near *KLHL35* might persist from birth until adulthood [32]. The 144 CpGs identified by Adkins et al. in offspring and 87 CpGs revealed by Moore et al. in adult daughters were both enriched in the processes associated with neurological regulation and metabolism, which implied that maternal age might affect the metabolism and neurodevelopment in later life of offspring [31, 33]. Our results also presented the DNA methylation changes involved in the regulation of neuron development, Rho protein signal transduction, glycosyl compound metabolic process, hexose metabolic process and catabolic process in AMA-offspring. This was consistent with the greater risks of neurological and neuropsychiatric diseases observed in AMA offspring in humans [63], and the abnormal hippocampal gene expression and impaired learning and memory observed in the AMA offspring of mice model [29, 64]. However, the controversies about the relationship between AMA and the metabolism disorders in the offspring[28] call for long-term follow-up research in larger cohorts. Another notable aspect was the potential disturbance of DNA methylation involved in the respiratory electron transport chain, mitochondrial gene expression and protein targeting to the ER, which were all related to oxidative stress, the key mechanism of endothelial dysfunction and arterial damage, which ultimately increased the risk of vascular disease and arterial stiffness [65]. In this context, our data supported the points proposed by previous AMA cohorts that AMA offspring are at greater risk of cardiovascular diseases due to oxidative stress.

Previous studies on the relationship between maternal age and the offspring DNA methylome are limited by the lack of maternal methylation data and the potential influence of confounding diseases, such as breast cancer and cleft lip [31-33]. Benefiting from our strict inclusion criteria and the availability of data from both parents and offspring, we were able to directly evaluate the intergenerational inheritance phenomenon of the effects of AMA on either the DNA methylome or transcriptome. Enrichment analysis for 109 common DEGs and 78 common DMRs between the mother and offspring groups suggested their participation in processes involving neuron differentiation, immune and protein modification. Meanwhile, the paired intergenerational analysis further confirmed the high relevance in special genes or genomic regions, and those intergenerationally correlated DEGs and DMRs represented various dynamic patterns during the development of oocyte and early embryo. The stage-specific DNA methylation patterns for those correlated DMRs supported the view that the drastic de novo DNA methylation happened in every stage, and DNA methylation reprogramming in preimplantation embryo development was shaped by the combination of the global demethylation and local remethylation [66]. It also implied that those DMRs might play key roles in regulating embryo development, which need further verification. Among those correlated DEGs, genes with the RO4 patterns showed quite striking changes during the preovulatory stage. *E**GR1*, a gene belonging to the EGR gene family, has been demonstrated to be induced by LH and encodes transcription factors important for ovulation in mice [42]. *CAPN3* may have a potential regulatory role in embryonic muscle fiber phenotype and development [67]. Those genes with RO3, RO6, and RO9 patterns might be involved in primordial follicular activation. The similarity of the dynamic pattern implied that other genes or regions within those clusters might exert similar functions in gametogenesis and embryonic development process, but further investigations are needed.

AMA-related transcriptional changes in oocytes were significantly enriched in the processes of mitochondrial function, oxidative stress and actin-binding alteration [53]. Consistent with this, AMA would also induce a significant global decline in the transcriptome of the blastocyst and repressed the processes related to mitochondrial function and the cell cycle [26]. Apart from those overlapping AMA-DEGs (*HTRA3, CNTNAP1, DNAH10, MED12L and BST2,* et al.) among adult blood, cord blood, oocyte and embryo data, AMA-related changed gene expression associated with the cell cycle and cell division was observed in both the mother and offspring groups. Combined with the potential disturbance of mitochondrial electron leakage and gene expression in offspring, as mentioned earlier, this observation underscores the direct intermediary role of oocyte and embryo as biological stages vulnerable to the influence of AMA. In particular, *HTRA3* and *MED12L* were both decreased intergenerationally correlated AMA-DEGs, with the former co-altered in AMA embryos and the latter existing in AMA oocytes. The deletion of *HtrA3* causes the placenta capillaries dysfunction and intra-uterine growth restriction (IUGR) in mice model [68]. Thus, the decrease of *HTRA3* in AMA embryos suggested a further investigation of placental abornomarties in AMA pregnancy. *MED12L* (Mediator Complex Subunit 12L*)* is the homolog of *MED12*, a classical maternal factor that the oocyte-specific ablation of which in mice would influence embryo development but not disrupt the folliculogenesis and ovulation [69]. The relatively high expression of *MED12L* was maintained until the 8-cell stage, and therefore implied that it may also serve as a maternal factor, the abnormal downregulation of which in AMA-oocyte may also disrupt the following early embryo development. The sharply increased expression of *CD24* in oocyte of preovulatory follicle suggests its important role in oocyte development. Previous study also verified that *CD24* is critical for triggering ovulation [43]. Although *CD24* did not observe in the AMA-related DEGs list of MII oocyte provided by Zhang, et.al [47], our unpublished data do suggest that the expression of *CD24* may also be disturbed in human AMA-oocyte. Taken together, this evidence indicated that some of the changes in the AMA offspring may be due to the abnormal changes in the gene expression network of oocytes and preimplantation embryos. Our results supported the explanations offered by the Developmental Origins of Health and Disease (DOHaD) hypothesis for the later-life health problems of AMA offspring [70].

Our research has some limitations. First, although previous research by Ronald M Adkins et al. found no significant relationship between maternal age and the methylation pattern reflecting blood cell populations, it would be ideal to separate individual cell populations for analysis or perform analysis based on the rapidly developing single-cell method. Second, studies in larger prospective cohorts will be helpful to validate our observations, to supplement the paired samples of nuclear families with rigorous exclusion criteria applied in our study. Third, only the cord blood was evaluated in our study, and the biopsy tissue from adult offspring and the extraembryonic tissues, such as the villus from the placenta, need to be further investigated for comprehensive and detailed recognition of the influence of AMA across organs and developmental stages. In conclusion, we systematically explored the impact of AMA on the DNA methylome and transcriptome of parents and offspring. AMA might induce intergenerational epigenetic and transcriptional changes involving immune and metabolism pathways, some of which were rooted in oocyte and early embryo, and then passed on to the next generation.

**4. EXPERIMENTAL PROCEDURES**

**4.1 Ethics**

All samples were collected after patients signed informed consent and in accordance with ethical standards. This research was approved by the Reproductive Study Ethics Committee of Peking University Third Hospital. (approval number scheme: 2016SZ-015).

**4.2 Sample collection and treatment**

Thirty nuclear families with fetuses (20 parents-offspring families and 10 mother-offspring families) were recruited in this research and were divided into the AMA group and the Young group based on maternal age. The mean age of AMA mothers was 39 ± 1.22 years, while that of young mothers was 29.8 ± 2.59 years. The BMI was not significantly different between AMA and Young mothers. Women with polycystic ovarian syndrome (PCOS), diabetes, hypertension, hyperthyroidism, hypothyroidism, systemic lupus erythematosus, or pregnancy complications such as preeclampsia, gestational diabetes mellitus, severe metabolic syndrome, or intrauterine infection were not eligible. Families with premature babies or infants who suffered from chromosomal abnormalities, any birth defects, or the Apgar score below 7 were also excluded. All women delivered by cesarean section at term (37 to 42 weeks). Approximately 4ml of umbilical cord blood and parental peripheral blood samples were collected on the day of delivery and treated within 2 h. Parental peripheral blood samples were collected before delivery in order to avoid any effects of blood transfusion during delivery. Of the 4 ml samples, 1ml was used for DNA extraction and 1ml was used for RNA extraction immediately, and the rest was stored at -80 ° C for backup.

**4.3 DNA extraction**

QIAamp® Blood Mini Kit (Qiagen Cat# 51104) was used to collect genomic DNA (gDNA) from the blood samples following the manufacturer's instructions. The gDNA was evaluated by a NanoDrop 300 ultraviolet spectrophotometer (ALLSHENG#AS-11020-00) to ensure an A260/A280 value ranged between 1.8 and 2.0, and stored in -80 ℃.

**4.4 RNA extraction**

QIAamp RNA Blood Mini Kit (Qiagen Cat# 52304) was used to extract total RNA from the whole blood sample. The RNA was evaluated a NanoDrop 300 ultraviolet spectrophotometer (ALLSHENG#AS-11020-00) and Agilent 2100 Bioanalyzer (Agilent# G2939BA). Qualified RNA samples were referred to as samples with an A260/A280 value of approximately 2.0 and an RNA intergrity number (RIN) of no less than 7. A total of 500ng of total RNA was reverse transcribed to obtain cDNA using the PrimeScript™ RT reagent Kit (TaKaRa Cat# RR047A). The remaining RNA sample was applied for library construction for mRNA-seq.

**4.5 Reduced representation bisulfite sequencing (RRBS) library construction and sequencing**

RRBS was performed as follows: The digestion of 3 ng unmethylated lambda DNA (Thermo Scientific, Cat# SD0021) and 500 ng of gDNA in 40 µl mixture systems was performed by adding 5 µl FastDigest MspI and 5µl 10x FastDigest buffer **(**Thermo Scientific Cat# FD0544). Then, the end-repair and adapter ligation of the digested DNA fragments were handled with the NEBNext Ultra DNA Library Prep Kit (NEB Cat# E7370), following the recommended instructions in the manual. Specifically, NEBNext methylated adapter (15 µM; NEB Cat# E7535) was used to prevent the change in adaptor sequence during bisulfite conversion treatment. After the digestion of U bases by 3 µl USER enzyme (NEB Cat# E6610A), size selection of DNA fragments was performed through agarose gel electrophoresis (2% TAE gel) and excision of gel slices containing targeted 200-700 bp DNA, which were extracted by a gel DNA recovery kit (VISTECH Cat# DC2005). Next, a MethylCode bisulfite conversion kit (Thermo Scientific Cat# MECOV-50) was used for bisulfite conversion according to the manuals. The converted fragments were amplified and tagged with specific barcode sequences by 12 cycles of PCR using Kapa HiFi U+ Master Mix (Kapa Biosystems Cat# KK2801). Finally, the product was purified by two rounds of cleanup using 0.8X Agencourt AMPure XP Beads (Beckman Cat# A63881), followed by quality testing in a Qubit 3.0 Fluorometer (Thermo Scientific Cat# Q33216). After the detection of exact fragment distributions and molar concentrations with a Fragment Analyzer™ Automated CE System (Analysis Kit: Cat# DNF-474-0500) and a Library Quant Kit for Illumina (NEB Cat# E7630L), the qualified DNA methylation libraries were sequenced on the Illumina NovaSeq platform with the PE150 strategy.

**4.6** **mRNA-seq library construction and sequencing**

mRNA libraries were generated using the NEBNext® UltraTM RNA Library Prep Kit for Illumina® (NEB Cat# E7530L) according to the manufacturer's recommendations. In short, poly(A) mRNA was isolated from approximately 500 ng total RNA with NEBNext Magnetic Oligo d(T)25 Beads provided in NEBNext Poly(A) mRNA Magnetic Isolation Module (NEB Cat #E7490). After fragmentation, priming, and the subsequent first-strand and second-strand cDNA synthesis, double-stranded cDNA was obtained and purified by 1.8X AMPure XP Beads (Beckman Cat# A63881). Then, the end preparation and adapter ligation of double-stranded DNA fragments were performed, and the hairpin loop structure within the adaptor was cut by incubating the product with USER Enzyme (NEB Cat# M5505L). Finally, purified adaptor-ligated DNA fragments were amplified and tagged with specific barcode sequences by PCR. The final mRNA libraries were assessed and sequenced as previously described in the RRBS protocol.

**4.7** **qRT-PCR (Real-time quantitative reverse transcription polymerase chain reaction)**

qRT-PCR for reversed cDNA was performed with PowerUp™ SYBR™ Green (Thermo Fisher Cat# A25742) in the QuantStudio 3 Real-Time PCR system as follows: 95 °C for 5 min, followed by 40 cycles of 95 °C for 30 s, 60 °C 40 s and 72 °C 1 min, followed by 72 °C 5 min. The delta-delta-Ct (^ΔΔ^Ct) algorithm was performed to calculate relative gene expression.  Each experiment was performed three times, and *ACTB* was used as the control. The primers are listed below：

***SCD*** (F: ACGCTTGTGCCCTGGTATTT/R: GCACCACAGCATATCGCAAG)

***CD24*** (F: GCTCCTACCCACGCAGATTTA/R: GACCACGAAGAGACTGGCTG)

***SLC28A3*** (F: TGTCAGCACCTGCGTCAT/R: CCTGCCATTCCACTCCC)

***HTRA3*** (F: CTGTGTTGTTGCTGGGTCAC/R: GTGTTCTGTAGGGCGAAGGG)

***MED12L*** (F: CTCCCTCAGTATCCAGGGCT/R: CTGCTGCAAAGGCATCTGTG)

***ACTB*** (F: CATGTACGTTGCTATCCAGGC/R: CTCCTTAATGTCACGCACGAT)

**4.8 Data downloading and processing**

The single-cell gene expression matrix for human oocytes during folliculogenesis and pre-implantation embryo was downloaded from our previously published datasets [38, 40]. The expression level was estimated using the fragments per kilobase million (FPKM). The scCOOL-seq data of human preimplantation embryos were downloaded from previously published datasets [41], and processed as previously described. Briefly, Bismark software (version 0.23.0) [71] was applied to align qualified reads to the Homo sapiens reference genome (human GRCh38/hg38) with the parameter "--paired-end and --non_directional”. Those unmapped reads were then re-aligned with single-end and non-directional model. After removing the PCR duplicates, the methylation levels of CpG sites were extracted using the function "bismark_methylation_extractor" in Bismark software (version 0.23.0) for downstream analysis. The aging and longevity genes list were downloaded from the Aging Atlas [35] and LongevityMap (Build 3) [34].

**4.9 Fundamental analysis for RRBS data**

All 150 bp bisulfite sequencing paired-end reads were trimmed to delete adaptors, bases of substandard quality (Q <20), and the reads shorter than 36 bases using TrimGalore software (https://www.bioinformatics.babraham.ac.uk/projects/trim_galore/; version 0.6.6). and Cutadapt (version 1.18), with the parameters " --quality 20 --phred33 --stringency 3 --length 36 --rrbs --paired --trim1". For bisulfite conversion rate evaluation, phage λ genome was used as an extra reference using the function "bismark_genome_preparation" in Bismark software (version 0.23.0) [71]. Bismark with the parameter “bowtie2" was used to map the clean reads to the spiked-in phage λ genome, and the bisulfite conversion rate was determined by the ratio of the number of unmethylated Cs to the total number of Cs detected. Sixty samples with a bisulfite conversion rate of more than 99% were retained for downstream analysis. Subsequently, Bismark with the parameter “bowtie2" was performed to align clean reads to the Homo sapiens reference genome (human GRCh38/hg38). Only the uniquely mapping readings with less than 2% mismatch were retained. Then, the number of reads supporting Cs and supporting Ts in each CpG site was counted using the function "bismark_methylation_extractor" in Bismark with the parameter " --paired-end --no_overlap". Finally, the coverage files recording the methylation state of CpG sites were inputted into the R package methylKit (version 1.10.0) for further analysis, and only CpG sites on autosomes with more than 5-fold read coverage were retained. The genome was tiled into consecutive 200 bp windows, and the 200 bp bins covered more than three CpG sites and existed in at least eight samples per group (AMA-offspring, AMA-mother, AMA-father, Young-offspring, Young-mother, and Young-father) were retained for downstream analysis. Detailed information including sequencing depth and bisulfite conversion rate, the number of covered CpG sites and retained bins, is provided in Supplementary Table **S2.**

**4.10 Copy number variation analysis**

For each RRBS library, the software “readCounter” in the HMMcopy suite following the R package “HMMcopy” (version 1.26.0) was applied to calculate the CNVs at 1 Mb resolution based on mapped RRBS reads sorted by SAMtools (version 1.3.1). The CNVs were plotted by the R function “points” and “plot”.

**4.11** **Global DNA methylation level estimation and differentially methylated regions (DMRs) identification**

The DNA methylation level of any retained 200 bp bin was calculated using the R package methylKit (version 1.10.0) [72] as the ratio of the total count of Cs and the total count of Cs and Ts bases within that bin. Based on this, the methylation level of each sample was calculated by averaging the DNA methylation levels of all bins. Intergroup comparisons between the AMA and Young groups were also performed using the R package methylKit (version 1.10.0) [72], and DMRs was referred as 200 bp bins with q-values no more than 0.05 and the mean methylation difference thresholds no less than 15%.

**4.12 DNA methylation pattern around the genic region**

The -15 kb upstream of the TSS and 15 kb downstream of the transcription end site (TES) of each gene were separately split into nonoverlapping 100 bp windows, while the gene body range from TSS to TES was equally divided into 100 fractions. The average DNA methylation levels within every window or faction were calculated, and the mean value of each genomic locations type was then computed to profile the global methylation pattern around the genic region for every sample. In addition, the mean value of all samples in the sample group was used to profile the global methylation pattern around the genic region for each group. The methylation pattern was visualized by the R function “plot”.

**4.13 DNA methylation levels within various genomic elements**

The coordinate files of the known genomic elements were acquired from the UCSC Genome Browser, including Low_complexity elements, CpG island (CGI), gene body, 5′-UTR, 3′-UTR, three types of promoters (high-CpG-density promoters (HCP), intermediate-CpG-density promoters (ICP), and low-CpG-density promoters (LCP)) as previously defined [73, 74], and repetitive elements classified into 7 categories: long terminal repeat (LTR), long interspersed elements (LINE), short interspersed elements (SINE), Retroposon_SVA, transposon, satellite, and microsatellite. The coordinate files of the imprinting control region (ICRs) were obtained from the paper of Hamada et al. [75]. Metastable epialleles (MEs) were obtained from the paper of Noah J. Kessler., et al. [76]. To evaluate the average DNA methylation level of each type of genomic element, only regions covering more than three CpG sites were reserved, and the mean methylation level of all retained CpG sites within a specific region was defined as the DNA methylation level of the corresponding region.

**4.14 DNA methylation pattern of selected DMRs in preimplantation embryos**

To calculate the methylation level of each intergenerationally correlated DMR mentioned in Supplementary Figure **S6** and **S7**, each DMR was expanded with additional 150 bp in both the upstream and downstream directions. Then, each single-cell DNA methylation data recording methylation level of every CpG sites was used as input, and expanded DMRs covering more than three CpGs in DNA methylome data of early embryo development was reserved. The DNA methylation level of corresponding DMRs was calculated by averaging the methylation level of all retained CpG sites within a specific expanded DMR. Next, samples with at least one DMRs of not NA value were reserved and merged into single file. The average methylation value of every sample in different stages was calculated respectively, and defined as the methylation level of the target expanded DMR in each stage. Ward hierarchical clustering for each intergenerationally correlated DMR was performed using the function “hclust” in R package stats (version 3.6.0) based on the scaled average DNA methylation level in different stages of early embryo development.

**4.15 RNA-seq data analysis**

The quality of the raw fastq data was first assessed by the FastQC tool(version 0.11.9), and then processed by the software TrimGalore with the parameter of “--quality 20 --stringency 3 --length 36 --paired” to remove the adapter, ploy-N, inferior -quality bases and reads less than 36 bases. Clean reads were then aligned to the Homo sapiens reference genome (human GRCh38/hg38) using STAR software (version 2.7.8a) with default parameters. The uniquely aligned reads were subsequently counted by featureCounts software (version 1.6.3) [77]. Finally, DEseq2 (version 1.24.0) was applied to generate the normalized count matrix for offspring or mother samples. Detailed information on all RNA-seq libraries is listed in Supplementary Table **S2**.

**4.16 Identification of differentially expressed genes (DEGs)**

The intergroup differential expression analysis for either offspring or mother was performed using the R package DESeq2 (1.24.1) [78]. Genes meeting the criteria of fold change values more than 1.5 and p values less than 0.05 were defined as DEGs.

**4.17 Principal component analysis (PCA) and hierarchical clustering analysis**

The average methylation value of the 200 bp bins around the entire genome was used for the hierarchical clustering analysis and principal component analysis (PCA) to evaluate the global DNA methylation similarity of offspring and parental samples, with the function “clusterSamples” in R package methylKit (version 1.10.0) [72] and the function “pca” in R package pcaMethods (version 1.76.0)[79], respectively. PCA was also performed to evaluate the transcriptome profile of offspring and mother samples with the function “pca” in the R package pcaMethods (version 1.76.0), using the normalized counts matrix for gene expression generated by DESeq2 (version 1.24.0). The distance matrices among RNA libraries were calculated using function dist with default paramenters in the R package stats (version 3.6.0) and were visualized using the R package pheatmap (version 1.0.12). The classification of the dynamic pattern of DNA methylation during the development of preimplantation embryos and the dynamic pattern of gene expression during folliculogenesis and preimplantation embryos was based on the hierarchical clustering analysis, employing the function “dist” in the R package stats (version 3.6.0) and the function “hcluster” in the R package amap (version 08-18) with the parameter “method= pearson”.

**4.18 Correlation analysis**

The mean value of DNA methylation level of each remaining 200 bp bins was calculated for each group (AMA-offspring, AMA-mother, AMA-father, Young-offspring, Young-mother, Young-father), and the correlation coefficients and confidence intervals between AMA and Young groups were calculated by the function “stat_cor” in the R package ggpubr (version 0.4.0) with default parameters [80]. The Spearman’s correlation coefficients and confidence intervals between parents and offspring samples were calculated using the function “cor.test” and “cor” in R package stats (version 3.6.0) for either the methylation level of selected DMRs or the expression level of selected DEGs. Only DMRs or DEGs with a p-value threshold of 0.05 and correlation Spearman’s correlation coefficients (R value) greater than 0.6 were defined as candidate intergenerational related DMRs or DEGs.

**4.19 Genomic functional annotation**

The nearest genes, genomic features and the distance to the transcriptional start site (TSS) of DMRs were annotated using the R package ChIPseeker (version 1.20.0) and Homo sapiens annotation package org.Hs.eg.db (version 3.8.2) [81]. The promoter was identified as the region from 3 kb downstream to 3 kb upstream of the TSS. The coordinate files of genomic elements were downloaded from the UCSC Genome Browser as mentioned in “***DNA methylation levels in specific genomic elements”***. The function “foverlaps” in R package data.table (version 1.14.0) was applied to identified the DMRs with at least 1-bp overlap with any genomic elements.

**4.20 Gene Ontology (GO) enrichment analysis**

GO enrichment analysis for biological processes was performed to assess the potential biological functions of selected DMRs and DEGs, using the “enrichGO” function in the R/Bioconductor package “clusterProfiler (3.8.1)” packages

**4.21 Statistical analysis**

An unpairedtwo-tailed t-test in GraphPad Prism (Version 9.2.0) was applied to determine the significance of differences in the intergroup comparison of other clinical features for either parents or progenies. Fisher's exact test in SPSS (version 26.0) was used to determine the significance of differences in the comparative analysis of sexes and modes of birth. The statistical significance of Gene Ontology (GO) term enrichment analysis was determined using a hypergeometric test in the R package clusterProfiler, and the p value was adjusted by the multiple test adjustment (Benjamini-Hochberg, BH).

For bar-dot plots of gene expression determined by qRT-PCR, the significance of differences between two groups was determined by unpaired two-tailed t test in GraphPad Prism (Version 9.2.0). For box-dot plots of DNA methylation, the significance of differences between two groups was determined by the Wilcoxon rank-sum test. The p value for the comparison among multiple groups was determined by the Kruskal-Wallis test. For a column graph of the proportion of bins with significantly differential DNA methylation levels for paternal or maternal samples, the chi-squared test was applied to calculate the significance of differences between all 200 bp bin groups and correlated AMA-DMRs of offspring groups. Unless otherwise stated, Spearman-based correlation tests were used to determine the correlation coefficient and p-value between any two groups (ns: P>=0.05; ∗: P<0.05; ∗∗: P<0.01; ∗∗∗: P<0.001).

**5 ACKNOWLEDGEMENTS**

We thank the patients who participated in this study. We are very grateful to Peng Yuan for his insightful comments on our paper. We are very indebted to Xi Chen, Qianying Guo and Yuqian Wang for their advice and discussion about our manuscript. We also thank Shuangyan Tan at Peking University Third Hospital for helping us communicate with patients. This work was supported by Ministry of Science and Technology of China, National Key R&D Program of China (No. 2018YFC1004500), the National Natural Science Foundation of China (No. 81730038) and Beijing Science and Technology Planning Project (Z191100006619085).

**6 CONFLICT OF INTEREST**The authors declare no competing interests.

**7 AUTHOR CONTRIBUTIONS**

L.H. carried out all the experiments and W.C. performed the data analysis; L.H. and W.C. wrote the manuscript; Y.M., M.Q., W.C. and L.H. collected the samples; M.Q., Q.L., R.Y., Y.W. and Y.Z. participated in the modification of the manuscript; Z.Y. provided advice on the data analysis, J.Q. and L.Y. conceived the study and designed the experiments.

**8 DATA AVAILABILITY STATEMENT**

The sequence data in this research are available at the National Genomics Data Center database (accession number: HRA001886; https://ngdc.cncb.ac.cn/gsa-human/s/b3WY4c81).

**9 REFERENCES**

1. Attali, E. and Y. Yogev, *The impact of advanced maternal age on pregnancy outcome.* Best Practice & Research Clinical Obstetrics & Gynaecology, 2021. **70**: p. 2-9.

2. Kim, Y.N., et al., *Maternal age and risk of early neonatal mortality: a national cohort study.* Sci Rep, 2021. **11**(1): p. 814.

3. da Costa, J.P., et al., *A synopsis on aging—Theories, mechanisms and future prospects.* Ageing research reviews, 2016. **29**: p. 90-112.

4. Khan, S.S., B.D. Singer, and D.E. Vaughan, *Molecular and physiological manifestations and measurement of aging in humans.* Aging cell, 2017. **16**(4): p. 624-633.

5. Lehallier, B., et al., *Undulating changes in human plasma proteome profiles across the lifespan.* Nature medicine, 2019. **25**(12): p. 1843-1850.

6. Frick, A.P., *Advanced maternal age and adverse pregnancy outcomes.* Best Practice & Research Clinical Obstetrics & Gynaecology, 2021. **70**: p. 92-100.

7. Magnus, M.C., et al., *Role of maternal age and pregnancy history in risk of miscarriage: prospective register based study.* Bmj, 2019. **364**: p. l869.

8. Perez, M.F. and B. Lehner, *Intergenerational and transgenerational epigenetic inheritance in animals.* Nature cell biology, 2019. **21**(2): p. 143-151.

9. Horvath, S. and K. Raj, *DNA methylation-based biomarkers and the epigenetic clock theory of ageing.* Nature Reviews Genetics, 2018. **19**(6): p. 371-384.

10. Du, Z., K. Zhang, and W. Xie, *Epigenetic reprogramming in early animal development.* Cold Spring Harbor Perspectives in Biology, 2022. **14**(6): p. a039677.

11. Guo, F., et al., *The transcriptome and DNA methylome landscapes of human primordial germ cells.* Cell, 2015. **161**(6): p. 1437-1452.

12. Zhu, P., et al., *Single-cell DNA methylome sequencing of human preimplantation embryos.* Nature Genetics, 2018. **50**(1): p. 12-19.

13. Szyf, M., *Nongenetic inheritance and transgenerational epigenetics.* Trends in molecular medicine, 2015. **21**(2): p. 134-144.

14. Liu, Y., M. Zhi, and X.J.A.R.R. Li, *Parental age and characteristics of the offspring.* Ageing Research Reviews, 2011. **10**(1): p. 115-123.

15. Monaghan, P., et al., *Intergenerational transfer of ageing: parental age and offspring lifespan.* Trends in Ecology Evolution, 2020. **35**(10): p. 927-937.

16. Milekic, M., et al., *Age-related sperm DNA methylation changes are transmitted to offspring and associated with abnormal behavior and dysregulated gene expression.* Molecular psychiatry, 2015. **20**(8): p. 995-1001.

17. Heffner, L.J.J.N.E.J.M., *Advanced maternal age–how old is too old.* N Engl J Med, 2004. **351**(19): p. 1927-9.

18. Mikwar, M., A.J. MacFarlane, and F.J.M.R.R.i.M.R. Marchetti, *Mechanisms of oocyte aneuploidy associated with advanced maternal age.* Mutation Research/Reviews in Mutation Research, 2020. **785**: p. 108320.

19. Chamani, I.J. and D.L.J.F.i.E. Keefe, *Epigenetics and female reproductive aging.* Frontiers in Endocrinology, 2019. **10**: p. 473.

20. Llonch, S., et al., *Single human oocyte transcriptome analysis reveals distinct maturation stage‐dependent pathways impacted by age.* Aging Cell, 2021. **20**(5): p. e13360.

21. Zhang, J.-J., et al., *Advanced maternal age alters expression of maternal effect genes that are essential for human oocyte quality.* Aging, 2020. **12**(4): p. 3950.

22. Reyes, J., et al., *Differing molecular response of young and advanced maternal age human oocytes to IVM.* Human Reproduction, 2017. **32**(11): p. 2199-2208.

23. Grøndahl, M., et al., *Gene expression profiles of single human mature oocytes in relation to age.* Human Reproduction, 2010. **25**(4): p. 957-968.

24. Barone, S., et al., *Chromosome missegregation in single human oocytes is related to the age and gene expression profile.* International journal of molecular sciences, 2020. **21**(6): p. 1934.

25. Steuerwald, N.M., et al., *Maternal age-related differential global expression profiles observed in human oocytes.* Reproductive biomedicine online, 2007. **14**(6): p. 700-708.

26. McCallie, B.R., et al., *Compromised global embryonic transcriptome associated with advanced maternal age.* Journal of assisted reproduction and genetics, 2019. **36**(5): p. 915-924.

27. Cooke, C.-L.M. and S.T. Davidge, *Advanced maternal age and the impact on maternal and offspring cardiovascular health.* American Journal of Physiology-Heart and Circulatory Physiology, 2019. **317**(2): p. H387-H394.

28. Velazquez, M., et al., *Advanced maternal age causes adverse programming of mouse blastocysts leading to altered growth and impaired cardiometabolic health in post-natal life.* Human Reproduction, 2016. **31**(9): p. 1970-1980.

29. Sampino, S., et al., *Pregnancy at advanced maternal age affects behavior and hippocampal gene expression in mouse offspring.* Journals of Gerontology Series A: Biomedical Sciences and Medical Sciences, 2017. **72**(11): p. 1465-1473.

30. Paczkowski, M., W. Schoolcraft, and R. Krisher, *Dysregulation of methylation and expression of imprinted genes in oocytes and reproductive tissues in mice of advanced maternal age.* Journal of assisted reproduction and genetics, 2015. **32**(5): p. 713-723.

31. Adkins, R.M., et al., *Parental ages and levels of DNA methylation in the newborn are correlated.* BMC medical genetics, 2011. **12**(1): p. 1-12.

32. Markunas, C.A., et al., *Maternal age at delivery is associated with an epigenetic signature in both newborns and adults.* PloS one, 2016. **11**(7): p. e0156361.

33. Moore, A.M., et al., *Persistent epigenetic changes in adult daughters of older mothers.* Epigenetics, 2019. **14**(5): p. 467-476.

34. Budovsky, A., et al., *LongevityMap: a database of human genetic variants associated with longevity.* Trends in Genetics, 2013. **29**(10): p. 559-560.

35. *Aging Atlas: a multi-omics database for aging biology.* Nucleic Acids Research, 2021. **49**(D1): p. D825-D830.

36. De Cecco, M., et al., *L1 drives IFN in senescent cells and promotes age-associated inflammation.* Nature, 2019. **566**(7742): p. 73-78.

37. Shakiba, N., et al., *CD24 tracks divergent pluripotent states in mouse and human cells.* Nature communications, 2015. **6**(1): p. 1-11.

38. Zhang, Y., et al., *Transcriptome landscape of human folliculogenesis reveals oocyte and granulosa cell interactions.* Molecular cell, 2018. **72**(6): p. 1021-1034. e4.

39. Yan, R., et al., *Decoding dynamic epigenetic landscapes in human oocytes using single-cell multi-omics sequencing.* Cell Stem Cell, 2021.

40. Yan, L., et al., *Single-cell RNA-Seq profiling of human preimplantation embryos and embryonic stem cells.* Nature structural & molecular biology, 2013. **20**(9): p. 1131-1139.

41. Li, L., et al., *Single-cell multi-omics sequencing of human early embryos.* Nature cell biology, 2018. **20**(7): p. 847-858.

42. Yuan, S., et al., *Age-associated up-regulation of EGR1 promotes granulosa cell apoptosis during follicle atresia in mice through the NF-κB pathway.* Cell Cycle, 2016. **15**(21): p. 2895-2905.

43. Dong, J.-p., et al., *CD24: a marker of granulosa cell subpopulation and a mediator of ovulation.* Cell death & disease, 2019. **10**(11): p. 1-12.

44. Sammar, M., et al., *Expression of CD24 and Siglec-10 in first trimester placenta: implications for immune tolerance at the fetal–maternal interface.* Histochemistry and cell biology, 2017. **147**(5): p. 565-574.

45. Ntostis, P., et al., *The impact of maternal age on gene expression during the GV to MII transition in euploid human oocytes.* Human Reproduction, 2022. **37**(1): p. 80-92.

46. Kawai, K., et al., *Parental age and gene expression profiles in individual human blastocysts.* Scientific reports, 2018. **8**(1): p. 1-10.

47. Zhang, J.-J., et al., *Advanced maternal age alters expression of maternal effect genes that are essential for human oocyte quality.* Aging (Albany NY), 2020. **12**(4): p. 3950.

48. Singh, H., Y. Endo, and G. Nie, *Decidual HtrA3 negatively regulates trophoblast invasion during human placentation.* Human reproduction, 2011. **26**(4): p. 748-757.

49. Bowden, M.A., et al., *HTRA3 expression in non-pregnant rhesus monkey ovary and endometrium, and at the maternal-fetal interface during early pregnancy.* Reproductive Biology and Endocrinology, 2008. **6**(1): p. 1-11.

50. Sauer, M.V., *Reproduction at an advanced maternal age and maternal health.* Fertility and sterility, 2015. **103**(5): p. 1136-1143.

51. Heijmans, B.T., et al., *Persistent epigenetic differences associated with prenatal exposure to famine in humans.* Proceedings of the National Academy of Sciences, 2008. **105**(44): p. 17046-17049.

52. Martin, E.M. and R.C. Fry, *Environmental influences on the epigenome: exposure-associated DNA methylation in human populations.* Annual review of public health, 2018. **39**: p. 309-333.

53. Cimadomo, D., et al., *Impact of maternal age on oocyte and embryo competence.* Frontiers in endocrinology, 2018. **9**: p. 327.

54. Sanada, F., et al., *Source of chronic inflammation in aging.* Frontiers in cardiovascular medicine, 2018. **5**: p. 12.

55. Hultman, C., et al., *Advancing paternal age and risk of autism: new evidence from a population-based study and a meta-analysis of epidemiological studies.* Molecular psychiatry, 2011. **16**(12): p. 1203-1212.

56. Yoshizaki, K., et al., *Paternal age affects offspring via an epigenetic mechanism involving REST/NRSF.* EMBO reports, 2021. **22**(2): p. e51524.

57. Maxwell, P.H., *What might retrotransposons teach us about aging?* Current genetics, 2016. **62**(2): p. 277-282.

58. Gatev, E., et al., *Autosomal sex-associated co-methylated regions predict biological sex from DNA methylation.* Nucleic Acids Research, 2021. **49**(16): p. 9097-9116.

59. Fischer, K.E. and N.C. Riddle, *Sex differences in aging: genomic instability.* The Journals of Gerontology: Series A, 2018. **73**(2): p. 166-174.

60. Hägg, S. and J. Jylhävä, *Sex differences in biological aging with a focus on human studies.* Elife, 2021. **10**: p. e63425.

61. Levine, M.E., et al., *Menopause accelerates biological aging.* Proceedings of the National Academy of Sciences, 2016. **113**(33): p. 9327-9332.

62. Chen, Z., et al., *Advanced maternal age causes premature placental senescence and malformation via dysregulated α‐Klotho expression in trophoblasts.* Aging Cell, 2021: p. e13417.

63. Tearne, J.E., *Older maternal age and child behavioral and cognitive outcomes: a review of the literature.* Fertility and Sterility, 2015. **103**(6): p. 1381-1391.

64. Li, D., et al., *Vitamin D supplementation in mice with advanced maternal age and cognitive function of the offspring.* American Journal of Translational Research, 2021. **13**(7): p. 7641.

65. Zhao, S., et al., *Interplay between oxidative stress, cyclooxygenases, and prostanoids in cardiovascular diseases.* Antioxidants & redox signaling, 2021. **34**(10): p. 784-799.

66. Zhu, P., et al., *Single-cell DNA methylome sequencing of human preimplantation embryos.* Nature genetics, 2018. **50**(1): p. 12-19.

67. Zhu, W., et al., *Expression of CAPN3 gene in skeletal muscles and its association with myofiber traits during embryonic and early post-hatching development in ducks.* Acta Veterinaria et Zootechnica Sinica, 2014. **45**(3): p. 385-390.

68. Li, Y., et al., *Maternal HtrA3 optimizes placental development to influence offspring birth weight and subsequent white fat gain in adulthood.* Scientific reports, 2017. **7**(1): p. 1-14.

69. Wang, X., et al., *Med12 regulates ovarian steroidogenesis, uterine development and maternal effects in the mammalian egg.* Biology of Reproduction, 2017. **97**(6): p. 822-834.

70. Suzuki, K., *The developing world of DOHaD.* Journal of developmental origins of health and disease, 2018. **9**(3): p. 266-269.

71. Krueger, F. and S.R. Andrews, *Bismark: a flexible aligner and methylation caller for Bisulfite-Seq applications.* bioinformatics, 2011. **27**(11): p. 1571-1572.

72. Akalin, A., et al., *methylKit: a comprehensive R package for the analysis of genome-wide DNA methylation profiles.* Genome biology, 2012. **13**(10): p. 1-9.

73. Hardarson, T., et al., *A morphological and chromosomal study of blastocysts developing from morphologically suboptimal human pre‐embryos compared with control blastocysts.* Human reproduction, 2003. **18**(2): p. 399-407.

74. Xie, W., et al., *Epigenomic analysis of multilineage differentiation of human embryonic stem cells.* Cell, 2013. **153**(5): p. 1134-1148.

75. Hamada, H., et al., *Allele-specific methylome and transcriptome analysis reveals widespread imprinting in the human placenta.* The American Journal of Human Genetics, 2016. **99**(5): p. 1045-1058.

76. Kessler, N.J., et al., *Establishment of environmentally sensitive DNA methylation states in the very early human embryo.* Science advances, 2018. **4**(7): p. eaat2624.

77. Liao, Y., G.K. Smyth, and W. Shi, *featureCounts: an efficient general purpose program for assigning sequence reads to genomic features.* Bioinformatics, 2014. **30**(7): p. 923-930.

78. Love, M.I., W. Huber, and S. Anders, *Moderated estimation of fold change and dispersion for RNA-seq data with DESeq2.* Genome biology, 2014. **15**(12): p. 1-21.

79. Stacklies, W., et al., *pcaMethods—a bioconductor package providing PCA methods for incomplete data.* Bioinformatics, 2007. **23**(9): p. 1164-1167.

80. Kassambara, A. and M.A. Kassambara, *Package ‘ggpubr’*. 2020.

81. Carlson, M., et al., *org. Hs. eg. db: Genome wide annotation for Human.* R package version, 2019. **3**(2).

**10 SUPPLEMENTARY DATA**

Supplementary data are available online.

**11 FIGURE LEGENDS**

**Figure 1** **Distinct AMA-related DNA methylation changes in the mother and father groups.** (A) Flowchart of the experiment and data analysis. (B) Three-dimensional scatter plot showing the distribution of parental samples in the four groups (AMA-mother, Young-mother, AMA-father, Young father). The first three principal components from principal components analysis (PCA) based on the 200 bp tiles DNA methylation pattern (n=701937) were used; sample size: n=40. (C) Box plot showing the distribution of DNA methylation level in four groups. Each dot represents the average DNA methylation level of each sample; the p-value between AMA and Young groups was determined by Wilcoxon rank-sum test. (ns: P>0.05); the p-value for the comparison among multiple groups was determined by Kruskal-Wallis test. (D) Mean DNA methylation levels along with the gene bodies, and 15 kilobases (kb) upstream of the transcription start site (TSS) and 15 kb downstream of the transcription end site (TES) of all genes. (E) Box plot presenting the distribution of the average DNA methylation level of specific genome elements in the AMA-mother group (red) and Young-mother group (blue); each dot represents the average DNA methylation level for each sample; the p value between the AMA and Young groups was determined by the Wilcoxon rank-sum test. (F) Heatmap showing the DNA methylation of AMA-DMR in the mother group (left) and father group (right). Blue bars represent the Young group, and red bars represent the AMA group. Orange bars represent hyper DMRs, while green bars represent hypo DMRs. (G) Venn diagram showing the numbers of overlapping and nooverlapped DMRs among the four groups; the corresponding relative genic location and nearby gene for each DMR in the targeted categories are presented on the left. Distal stands for distal intergenic. (H-I) Venn diagram showing the number of intersections between genes provided by the genAge database and genes near AMA-DMRs in either the mother groups (H) or the father group (I). (J) Venn diagram showing the number of intersections between genes near AMA-DMRs and SASP genes in the mother groups. The relative genic locations and concrete genomic coordinates for the corresponding DMRs of overlapping genes are presented on the right. (ns: P>0.05, ∗P<0.05; ∗∗P<0.01, ∗∗∗P<0.001).

**Figure 2 AMA-related DNA methylation changes in offspring.** (A) Three-dimensional scatter plot showing the distribution of neonatal samples in the AMA and Young groups (sample size: n = 20); the first three principal components of PCA based on the 200 bp tiles DNA methylation pattern (n= 701937) were used. (B) Box plot showing the distribution of the mean DNA methylation level in the AMA and Young groups; each dot represents the average DNA methylation level of the corresponding sample; the p value between the AMA and Young groups was examined by the Wilcoxon rank-sum test. (ns: P>0.05). (C) Average DNA methylation levels of the gene bodies, and 15 kb upstream of the TSS and 15 kb downstream of the TES of all genes for each sample. (D) Box plot showing the distribution of the average DNA methylation level of specific genome elements as mentioned in **Figure 1E** in the AMA (red) and Young groups (blue); each dot represents the average DNA methylation level for each sample; the p value between the AMA and Young groups was determined by the Wilcoxon rank-sum test. (E) Heatmap showing the DNA methylation level in the AMA-DMRs of the AMA offspring group and Young offspring group. Blue bars represent the Young group, and red bars represent the AMA group. The orange bars represent the hyper DMRs, while the green bars represent the hypo DMRs. (F) Column graph showing the proportion of AMA-DMRs in different genomic features. (G) Venn diagram showing the intersection of genes provided by the genAge database and genes near AMA-DMRs. (H) Venn diagram showing the intersection of genes near AMA-DMRs and SASP genes. The relative genic locations and concrete genomic coordinates for the corresponding DMRs of overlapping genes are presented on the right. (I) UpSet plot revealing the relationship among six lists of AMA-DMRs in father groups, mother groups and offspring groups. Bar plots on the left and top represent the number of DMRs in the corresponding group. MK-common DMRs are DMRs identified as common between the mother and offspring groups; FK-common DMRs are DMRs identified as common between the father and offspring groups; FMK-common are DMRs identified as common among the mother, father and offspring groups.

**Figure 3 AMA-related transcriptional alterations in the mother and offspring.** (A-B) Volcano plot simultaneously displaying the p-value and the fold change in gene expression level in the comparison between the AMA and Young groups for either the mother (**A**) or offspring (**B**) groups. Red dots and blue dots represent the upregulated and downregulated DEGs identified between the AMA group and Young group; light grey dots refer to genes with no significant change. (C) Venn diagrams displaying the number of overlapping or no-overlapping AMA-DEGs between the mother and offspring groups (left), and the number of intersections between SASP genes and common DEGs in mother and offspring groups (right). (D) Bubble chart showing representative Gene Ontology (GO) terms for common AMA-DEGs mentioned in (**C**). The gene number in all enrichment terms was not less than three, with a p-value < 0.05. The p-value was measured by hypergeometric test. (E-G) Heatmap showing the DNA methylation level of AMA-DMRs whose nearest genes belonged to AMA-DEGs in either the offspring group **(E)** or in the mother group **(G, left)** or the gene expression level of AMA-DEGs overlapped with genes near AMA-DMRs in either the offspring group **(F)** or in the mother group **(G, right).** Blue bars indicate the Young group, and red bars indicate the AMA group. Gray and orange bars indicate Up-DEGs overlapping with genes near hypo DMRs and genes near hyper DMRs, respectively. Green and purple bars indicate Down-DEGs overlapping with genes near hypo DMRs and genes near hyper DMRs, respectively. (H) Box diagram showing the gene expression level of *SLC28A3* in the four groups (AMA-Offspring, Young-Offspring, AMA-mother, Young-mother). (I) Column diagram displaying the relative gene expression level of *SLC28A3* determined by qRT-PCR in the AMA and Young groups for either neonatal samples (left) or maternal samples (right). Each dot represents the relative gene expression level of each sample; error bars refer to the standard deviation. The p value between the AMA and Young groups was determined by unpaired t-test. (∗: P<0.05).

**Figure 4** **Intergenerational correlation of AMA-related changes in either the DNA methylome or transcriptome.** (A) Column diagram displaying the relative gene expression levels of *CD24*  determined by qRT-PCR in the AMA and Young groups for either neonatal samples or maternal samples. Each dot represents the relative gene expression level of each sample. Error bars refer to the standard deviation. The p value between AMA and Young groups was calculated by unpaired t test. (∗: P<0.05; ∗∗: P<0.01; ∗∗∗: P<0.001). (B) Scatter diagram showing the relative gene expression level determined by qRT-PCR for *CD24* in paired maternal and neonatal samples. The blue straight line is the fitted linear regression line. Each dot represents a family. AMA families are shown in red, and Young families are in blue. The correlation coefficient and p value between the mother and offspring groups were calculated by Spearman-based correlation tests. The shadow indicates the 0.95 confidence level interval around the fitted linear regression line; n = 10. (C) Bar graph showing the number of intergenerational and no-intergenerational correlated DMRs in either MK-common DMRs or FK-common DMRs identified in **Figure 2I**, as well as the number of intergenerational and no-intergenerational correlated DEGs in MK-common DEGs as mentioned in **Figure 3C**. (D) Heatmap (right) showing the raw average DNA methylation level in different stages of early embryo development for each intergenerationally correlated DMR. Heatmap (left) and line graph showing the scaled average DNA methylation level in different stages of early embryo development for each intergenerationally correlated DMRs. Each DMR was expanded to an additional 150 bp upstream and downstream. Only expanded DMRs covering more than three CpGs in DNA methylome data of early embryo development were analyzed. Red dot in each stage refer to the median value of the scaled DNA methylation level of different DMRs. K-means clustering based on the methylation dynamic pattern was performed to cluster those DMRs. (E) Heatmap and line graph showing the scaled average gene expression level in different stages of human oogenesis for each detected intergenerationally correlated DEGs. Red dot in each stage referred to the median value of scaled gene expression level of different DEGs. K-means clustering based on the expression pattern was applied to classify those genes. (F) Heatmap showing the scaled gene expression level in each cell during early embryo development for each detected intergenerationally correlated DEG. K-means clustering based on the expression pattern is applied to classify those genes. Curve chart in the right showing the loess-smoothed row-scaled expression dynamics pattern for genes in selected six different clusters. The shadow denotes the 0.95 confidence level interval around the fitting curves. For specific cluster, each point refers to the median of row-scaled expression value of DEGs in each cells.

**Figure 5 Interrelationship among AMA-related transcriptional alterations in the mother, offspring, MII oocyte and blastocyst.** (A) Venn diagrams displaying the number of intersections for five lists of AMA-related DEGs from our maternal blood data, cord blood data, one published MII oocyte data and two published cry blastocyst data. Bar graph showing the total number of DEGs in each dataset. The top panel shows upregulated DEGs, while the bottom panel shows downregulated DEGs. (B) Scatter diagram showing the gene expression levels of *HTRA3* in paired maternal and neonatal samples. The blue straight line refers to the fitted linear regression line. The correlation coefficient and p value between the mother and offspring groups were calculated by Spearman-based correlation tests. (C) Box diagram showing the gene expression of *HTRA3* in four groups (AMA-Offspring, Young-Offspring, AMA-Mother and Young-Mother) (D) Line diagram showing the gene expression dynamics of *HTRA3* during oogenesis and early embryo development. (E) Scatter diagram showing the relative gene expression level determined by qRT-PCR for *HTRA3* in paired maternal and neonatal samples. The blue straight line is the fitted linear regression line. Each dot represents a family. AMA families are shown in red, and Young families are in blue. The correlation coefficient and p value between the mother and offspring groups were calculated by Spearman-based correlation tests. The shadow indicates the 0.95 confidence level interval around the fitted linear regression line; n = 10. (F) Column diagram displaying the relative gene expression levels of *HTRA3* determined by qRT-PCR in the AMA and Young groups for either neonatal samples or maternal samples. Each dot represents the relative gene expression level of each sample. Error bars refer to the standard deviation. The p value between AMA and Young groups was calculated by unpaired t test. (∗: P<0.05; ∗∗: P<0.01; ∗∗∗: P<0.001). (G) A schematic illustration showing the close connection between mother and offspring for the AMA-related alterations in either transcriptome or DNA methylome. The AMA-related alteration in three genes：*SLC28A3*, *CD24* and *HTRA3* represents three typical patterns for intergenerationally correlated AMA-DEGs mentioned in our study, respectively. AMA's impact on pregnant women will induce abnormal fluctuations in specific gene expression (such as *HTRA3*) in MII oocytes or preimplantation embryos, thus contributing to the similar changes observed in offspring. (H) A schematic diagram showing the interrelationship between AMA, AMA-related maternal molecular change, AMA-related offspring molecular change, and disease risk. AMA induced adverse changes on the mother and resulted in the increased risk of pregnant complications. The inheritable part of maternal changes, together with paternal impact and other external factors, lead to abnormal molecular changes in offspring and further influence their long-term health.

**Supplemental Figure S1 Quality assessment for each RRBS library.** (A) Copy number variations analysis of all analyzed samples. Each dot indicates a fragment detected at 1 million bp resolution. Green, blue, and red indicate normalized copy number values of 1 (loss), 2 (diploid) and 3 (gain). (B) Column diagram showing the total number of 200 bp bins covering at three CpG sites in each sample (top for bins without any filtration, bottom for bins remaining in at least 80% of the samples)

**Supplemental Figure S2 DNA methylation pattern of each sample.** (A) Scatter plot showing the distribution of all samples (sample size: n=60); the first two principal components of principal components analysis (PCA) based on the 200bp-tiles DNA methylation pattern (n= 701937) were used. (B) Hierarchical clustering for samples in the mother (top), father (middle) and offspring(bottom) based on the DNA methylation level of the remaining 200 bp bin. (C) Scatter plot showing the correlations of genome-wide methylation levels between AMA groups and Young groups for mother (top), father (middle) and offspring (bottom) respectively. The Pearson correlation coefficients (R) and statistical significance (p value) were computed and presented on the top right conner of each panel. The X-axis indicates the average methylation level of the AMA groups; the Y-axis shows the average methylation level of the Young groups; (D) The violin plot shows the distribution of DNA methylation levels for each sample in the mother (top), father (middle) and offspring (bottom). (E) Mean DNA methylation levels along the gene bodies, and 15 kb upstream of the TSS and 15 kb downstream of the TES of all genes for each parental sample. (F) Box plot presenting the distribution of the average DNA methylation level of specific genome elements in the AMA father group (red) and the Young father group (blue); each dot represents the average DNA methylation level for each sample; the p-value between the AMA and Young groups was determined by Wilcoxon rank-sum test. (G) Mean DNA methylation levels along gene bodies, and 15 kb upstream of the TSS and 15 kb downstream of the TES of all genes for each neonatal sample.

**Supplemental Figure S3 Distribution of AMA-related differential methylation values and DMRs.** (A-I) Histogram and density plots displaying the distribution of differential values of DNA methylation levels between the AMA and Young groups for the mother (A), father (D) and offspring (G) groups, respectively; hyper refers to the bins with increased DNA methylation levels in AMA group; hypo refers to the bins with decreased DNA methylation levels in the AMA group. Pie chart showing the proportion of hyper-DMRs and hypo-DMRs for mother (B), father (E) and offspring(H) groups, respectively; Box plot displaying the distribution of the average methylation level of either hyper-DMRs (left) and hypo-DMRs (right) for the mother (C), father (F) and offspring (I) groups, respectively. The p-value between the AMA and Young groups was determined by the Wilcoxon rank-sum test. (ns: P>0.05). (J) Circos plot and rainfall plot showing the distribution across the human genome (chromosome 1-22) for DMRs identified in the father, mother and neonatal groups. (K) Column graph showing the proportion of AMA-DMRs in different genomic features. The father and mother group are shown on the top and bottom, respectively.

**Supplemental Figure S4 Biological functions influenced by AMA-related methylation changes.** (A) Pie chart showing the proportion of AMA-DMRs located in or outside specific genomic elements for the offspring (top), mother (middle) and mother (bottom) groups, respectively. (B-C) Bubble chart showing selected biological process GO terms for nearest genes of AMA-DMRs identified in two parental groups (B) or offspring group (C). The gene number in all enrichment terms was not less than three, with a p-value < 0.05. The p-value was determined by hypergeometric test.

**Supplemental Figure S5 Overview of AMA-related transcriptional changes.** (A) Column diagram showing the number of genes detected in all samples (B) Heatmap showing the Euclidean distance among all samples (C) Scatter plot presenting the distribution of all samples (sample size: n=20); the first two principal components of PCA based on gene expression were used. (D) Heatmaps showing the expression level of AMA-DEGs identified in the offspring group (top) and mother groups (bottom). (E) Venn diagram showing the number of intersections between genes provided by the genAge database and AMA-DEGs identified in either the offspring group or mother group. (F) Bubble chart showing selected biological process GO terms for AMA-DEGs identified in offspring group and mother group. The gene number in all enrichment terms was not less than three, with a p-value < 0.05. The p-value was determined by hypergeometric test.

**Supplemental Figure S6 Intergenerational correlations for DNA methylation changes between father and offspring.** (A) Scatter diagram showing the DNA methylation of 200 bp bins in paired paternal and neonatal samples. The bins shown here all belonged to AMA-DMRs identified in offspring group, with a Spearman correlation coefficient (R) greater than 0.6 and p-value no less than 0.05. The blue straight line is the fitted linear regression line. The correlation coefficient and p value between the mother and offspring groups were calculated by Spearman-based correlation tests. (B) Column graph showing the proportion of 200 bp bins with or without significantly differential DNA methylation levels between the AMA and Young groups for paternal samples (no_sig: q-value >= 0.05; sig: q-value < 0.05). Left for all 200 bp bins remained in father groups, and right for correlated AMA-DMRs between father and offspring groups as shown in (A). Q-values for each 200 bp bins in the father group were calculated as mentioned earlier. The chi-squared test was applied to calculate the p-value between two groups (all 200 bp bins versus correlated AMA-DMRs). (C) Histogram and density distribution plot showing the distribution of the differential value of DNA methylation levels between the AMA and Young groups for correlated AMA-DMRs shown in (A). Hyper and hypo refer to the bins with increased or decreased DNA methylation levels in the AMA group, respectively.

**Supplemental Figure S7 Intergenerational correlations for DNA methylation changes between mother and offspring.** (A) Scatter diagram showing the DNA methylation of 200 bp bins in paired maternal and neonatal samples, similar to what was performed in Supplemental Figure S6A. (B) Column graph showing the proportion of 200 bp bins with or without significantly differential DNA methylation levels between the AMA and Young groups for maternal samples (no_sig: q-value >= 0.05; sig: q-value < 0.05). Left for all 200 bp bins remained in the mother groups, and right for correlated AMA-DMRs between the mother and offspring groups as shown in (A). q-values for each 200bp bins in mother group was calculated as mentioned in **section 4.9**. Chi-squared test was applied to calculate the p value between two groups (all 200bp bins versus correlated AMA-DMRs). (C) Histogram and density distribution plot showing the distribution of the differential value of DNA methylation level between AMA and Young groups for correlated AMA-DMRs shown in (A). Hyper and hypo refer to the bins with increased or decreased DNA methylation level in the AMA group, respectively. (D) Venn diagrams displaying the number of intersections among intergenerationally correlated DMRs shown in Supplemental Figure S6A, intergenerational correlated DMRs showing in Supplemental Figure S7A, and three lists of AMA-DMRs identified in the offspring, father, and mother groups, respectively.

**Supplemental Figure S8 Intergenerational correlations of transcriptional changes between mother and offspring.** (A) Scatter diagram showing the gene expression level of each mother-offspring correlated AMA-DEG in paired paternal and neonatal samples. Mother-offspring correlated AMA-DEGs referred to common AMA-DEGs identified in both the mother and offspring group, with a correlation coefficient (R) greater than 0.6 and p value no less than 0.05. The blue straight line refers to the fitted linear regression line. The correlation coefficient and p value between mother and offspring groups were calculated by Spearman-based correlation tests. (B) Line diagram showing the gene expression dynamics of *CD24* during oogenesis and early embryo development. (C) Column diagram displaying the relative gene expression level of *MED12L* determined by qRT-PCR in the AMA and Young groups for either neonatal samples or maternal samples. Each dot represents the relative gene expression level of each sample. Error bars refer to standard deviation. The p value between the AMA and Young groups was determined by unpaired t-test. (ns: P>=0.05;∗: P<0.05; ∗∗: P<0.01; ∗∗∗: P<0.001). (D) Scatter diagram showing the relative gene expression level determined by qRT-PCR for *MED12L* in paired maternal and neonatal samples. The blue straight line is the fitted linear regression line. The correlation coefficient and p value between the mother and offspring groups were calculated by Spearman-based correlation tests. The shadow indicates the 0.95 confidence level interval around the fitted linear regression line; n = 10.
